# Tissue rigidity phase transition shapes morphogen gradients

**DOI:** 10.1038/s41556-026-01954-4

**Published:** 2026-05-14

**Authors:** Camilla Autorino, Diana Khoromskaia, Louise Harari, Elisa Floris, Harry Booth, Cristina Pallares-Cartes, Vesta Petrasiunaite, Michael Dorrity, Bernat Corominas-Murtra, Zena Hadjivasiliou, Nicoletta I. Petridou

**Affiliations:** 1 Developmental Biology Unit, European Molecular Biology Laboratory, Heidelberg, Germany; 2 Collaboration for joint PhD degree between EMBL and Heidelberg University, Faculty of Biosciences, Heidelberg, Germany; 3 Mathematical and Physical Biology Laboratory, The Francis Crick Institute, London, UK; 4 Department of Physics and Astronomy, University College London, London, UK; 5 London Centre for Nanotechnology, University College London, London, UK; 6 Institute for Theoretical Physics, University of Münster, Münster, Germany; 7 Department of Biology, University of Graz, Graz, Austria

**Keywords:** Mesoderm, Gastrulation, Biophysics, Body patterning

## Abstract

During development, local mechanochemical cues within the cell microenvironment are translated into signalling pathways that drive cell fate decisions. Yet, as cells differentiate collectively, how global tissue-level properties shape these instructive cues remains unclear. Here we show that a tissue-scale rigidity transition guides patterning by tuning the length scales and timescales of morphogen signalling. By combining rigidity percolation theory, reaction–diffusion modelling, quantitative imaging and optogenetics in zebrafish, we uncover dynamical global tissue rigidity patterns that actively shape the Nodal morphogen gradient by locally changing its concentration and accelerating its signalling activity. In this self-generated mechanism, Nodal, besides instructing meso-endoderm fate specification, increases cell–cell adhesion strength via regulating planar cell polarity genes. Once the adhesion strength reaches a critical point, it triggers a rigidity transition which, in turn, induces the collapse of tissue porosity. The abrupt tissue reorganization negatively feeds back on Nodal signalling, impacting both its length scales, by restricting Nodal diffusivity, and its timescales, by speeding up the expression of its antagonist Lefty, thereby ensuring timely signal termination and robust patterning. Overall, we uncover a multiscale regulatory mechanism by which positional information and tissue material properties dynamically tune one another.

## Abstract (web summary)

Autorino et al. report a feedback loop between Nodal signalling and tissue phase transitions in zebrafish embryos. Nodal alters adhesion, triggering tissue rigidification and reduced porosity, which limits its diffusion and speeds up Lefty expression.

## Main

Embryo development is driven by processes spanning levels of biological organization. Accordingly, cell fate decisions, while guided by local mechanochemical signals generated at the level of the cell, may integrate information conveyed by properties arising at the supracellular or collective level, such as tissue rigidity, pressure or geometry^1–4^. How the cellular macroenvironment impacts cell fate decisions is still elusive, largely owing to the lack of approaches integrating tissue properties from a collective standpoint.

Recent work has grounded the shift between collective tissue states, for example, solid-like and fluid-like, to theoretical frameworks of material-phase transitions^5–9^. During phase transitions, the tissue collective material state changes abruptly when a smoothly varying cell control parameter, such as cell–cell adhesion or cell shape, crosses a specific value, the ‘critical point’^10–18^. In turn, tissue material properties are ultimately guided by the developmental programmes of cell fate specification, such as morphogen signals^14,19–22^. For instance, tissue rheological measurements in zebrafish embryos showed that Nodal signalling together with non-canonical Wnt signalling promote tissue solidification^14,19^, whereas in the avian skin, FGF and BMP signalling promote tissue solidification and fluidization, respectively^21^. Conversely, morphogen transport itself may be influenced by changes in the tissue architecture that defines the geometry of the extracellular space where morphogens diffuse or are taken up by cells^23–29^. If and how morphogen signalling and dynamic switches in tissue organization are coregulated and what are the functional implications of such an interplay remain open questions.

**Fig. 1:**
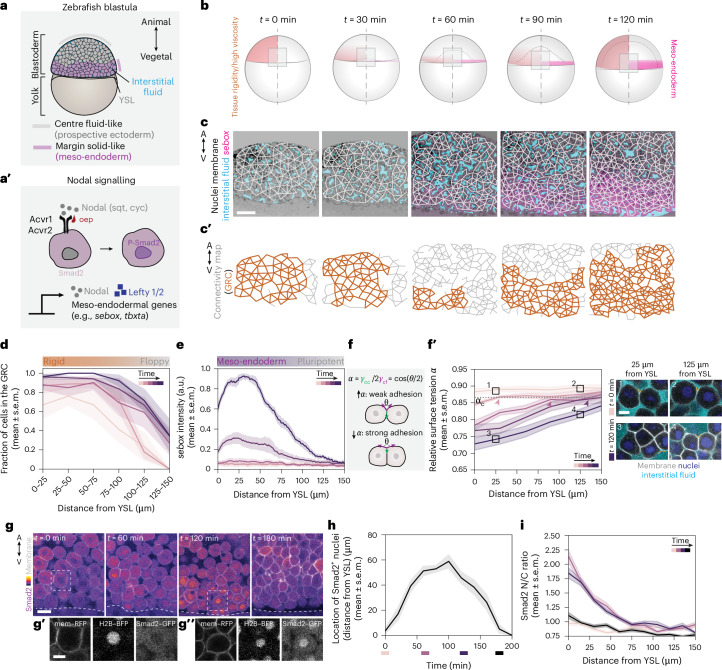
Meso-endodermal patterning in zebrafish correlates spatiotemporally with a tissue rigidity phase transition. **a**, Schematic diagram of the zebrafish embryo at blastula stage, composed of the blastoderm, yolk and YSL. The blastoderm is composed of loosely-attached cells surrounded by interstitial fluid (cyan). The meso-endodermal layer is at the blastoderm margin. **a’**, Schematic diagram of cell response to Nodal signalling, including phosphorylation of Smad2/3, translocation to the nucleus and transcription of *nodal*, *lefty* and meso-endodermal genes. **b**, Schematic diagram of the zebrafish embryo during meso-endoderm specification (right), coinciding with changes in blastoderm rigidity and viscosity (left). **c**, Exemplary projected confocal sections from timelapse of transgenic embryos expressing eGFP in meso-endodermal progenitor cells (Tg(mezzo:eGFP), *sebox*)) labelled with membrane–RFP, H2B–BFP and dextran-647 in the interstitial fluid, with overlaid connectivity maps. **c’**, Rigidity analysis of the cellular networks shown in **c**, with the GRC shown in dark orange. **d**,**e**, Plots of the distribution of the GRC (**d**) and *sebox* intensity (**e**), as a function of the distance from the YSL over time, at time intervals of 30 min. The shaded bars indicate the rigid–floppy boundary in **d** and the meso-endoderm–prospective ectoderm boundary in **e**. Data are presented as mean ± s.e.m. (*n* = 4 embryos for **d**, *n* = 8 embryos for **e**). **f**, Schematic diagram of the relative surface tension *α* as defined by the Young–Dupré relation, measured by the contact angle. **f’**, Plot of the distribution of *α* as a function of the distance from the YSL over time (left), at time intervals of 30 min, and exemplary confocal images of the contact angles (right) in the different blastoderm regions and timepoints (regions 1 and 3: cells close to YSL; regions 2 and 4: cells further away from YSL). Data are presented as mean ± s.e.m. (*n* = 8 embryos). **g**, Exemplary 2D confocal sections from live embryos labelled for membrane–RFP, H2B–BFP and Smad2-GFP. The dotted line indicates the YSL. Dotted boxes indicate close-ups at timepoints *t* = 0 min (**g’**) and at *t* = 120 min (**g”**). **h**, Plot of the distribution of nuclear Smad2-positive cells across the A–V axis as a function of time. Shaded boxes indicate the timepoints shown in **g**. Data are presented as mean ± s.e.m. (*n* = 6 embryos). **i**, Plot of the Smad2–GFP N/C ratio intensity over time as a function of the distance from YSL (*n* = 3 embryos), at time intervals of 60 min. Data are presented as mean ± s.e.m. Scale bars, 50 μm (**c**), 10 μm (**f’**, **g’** and **g”**), 20 μm (**g**). Source data

**Extended Data Fig. 1:**
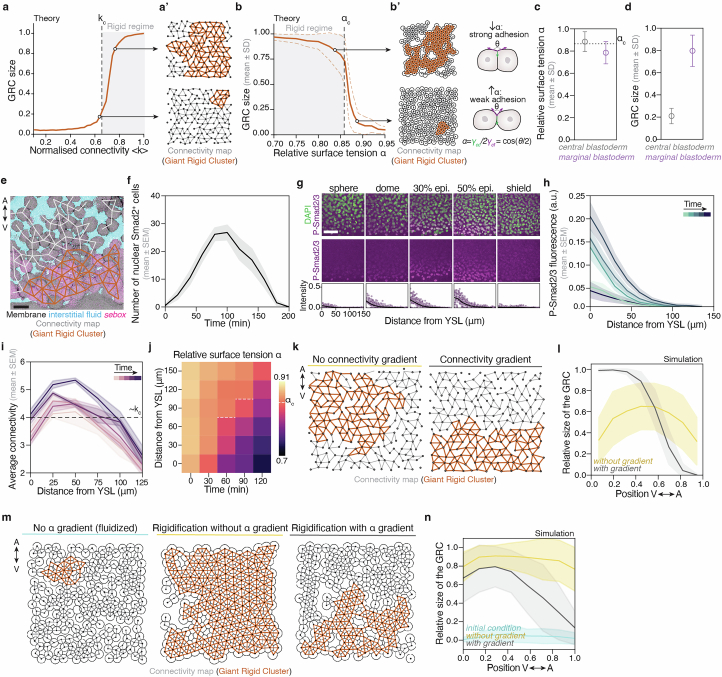
Dynamics of adhesion- and connectivity-dependent tissue rigidity transition during meso-endodermal fate specification in zebrafish. (**a**) Plot of the fraction of the network occupied by the GRC as a function of normalised connectivity <*k*> for simulated random networks and (**a**’) exemplary networks with connectivity above and below the critical point (adapted from^14^). Rigidity emerges at *k*_*c*_. (**b**) Plot of the fraction of the network occupied by the GRC as a function of the relative surface tension *α* and (**b**’) numerical simulations and overlaid connectivity and rigidity maps of cell arrays with *α* below or above *α*_*c*_ (adapted from^16^). Data are presented as mean ± SD. Rigidity emerges at *α*_*c*_. (**c, d**) Plots of the relative surface tension *α* (c) and GRC (d) in the central vs marginal blastoderm at the onset of epiboly (n = 3 embryos each)^14^. Data are presented as mean ± SD. (**e**) Exemplary confocal section from a transgenic embryo for the meso-endodermal marker *sebox* (Tg(mezzo:eGFP)) labelled with membrane-RFP and dextran-647 in the interstitial fluid at t 90 min, with overlaid connectivity and rigidity map, showing the border of the rigid domain coinciding with the border of the specification zone. (**f**) Plot of the number of nuclear Smad2-positive cells as a function of time for the experiments shown in Fig. 1g in a 180 μm wide region. Data are presented as mean ± SEM (n = 6 embryos). (**g**) Representative MIPs of fixed embryos stained for DAPI and P-Smad2/3 from blastula to gastrula stages (top) and exemplary quantifications of the P-Smad2/3 signal intensity normalised to DAPI as a function of the distance from YSL of the embryos shown above (bottom). (**h**) Same quantification as in (g) but averaged over several embryos for the reported stages. Data are presented as mean values ± CI (sphere n = 13, dome n = 9, 30%-epiboly n = 14, 50%-epiboly n = 16, shield n = 14 embryos). (**i**) Plot of the distribution of average connectivity as a function of the distance from the YSL, over time. Data are presented as mean ± SEM (n = 4 embryos). The dashed line indicates *k*_*c*_ for large networks. (**j**) Heat map of the relative surface tension *α* values of *wild-type* embryos over time and space. White dashed line shows crossing of *α*_*c*_ in space (n = 8 embryos). (**k**) Exemplary simulations showing GRC location without and with a gradient in connectivity along the A-V axis. (**l**) Plot of the GRC distribution as a function of the A-V axis for the networks shown in (k). Data are presented as mean ± SD. (**m**) Exemplary simulations of cell arrays with overlaid connectivity and rigidity maps. Simulations were performed starting with connectivity below *k*_*c*_ and at *α* ≈ 0.95, above *α*_*c*_ (left) and, then, decreasing *α* either without (middle) or with a gradient (right) along the A-V axis. As in the case of hard spheres (blue line), at high *α* regimes the GRC is negligible. (**n**) Plot of the GRC distribution as a function of the A-V axis for the conditions shown in (m). Data are presented as mean ± SD. The time axis in (h) corresponds to the stages in (g) light colour, sphere; dark colour, shield. The time axis in (i) corresponds to intervals of 30 min. A-V, Animal-Vegetal axis; GRC, Giant Rigid Cluster; YSL, Yolk Syncytial Layer. Scale bars: 20 μm (e), 50 μm (g). Source data

We explore these questions in the early zebrafish embryo, where spatiotemporal variations in blastoderm tissue viscosity coincide with its exit from pluripotency^19^, with the latter being driven by the Nodal morphogen^30^. At the blastula stage, the blastoderm is composed of pluripotent cells surrounded by interstitial fluid (Fig. 1a,b; *t* = 0 min). The first patterning event occurs at the onset of epiboly, when Nodal ligands, synthesized in the yolk syncytial laye (YSL), are secreted in the blastoderm forming a gradient along the animal–vegetal (A–V) axis^31–33^ (Fig. 1a,a’). When the marginal cells receive Nodal, Smad2/3 gets phosphorylated and enters the nucleus^34,35^ to initiate the transcription of meso-endodermal genes, Nodal itself and its inhibitor Lefty^33,36,37^ (Fig. 1a’), defining the specification region of the meso-endodermal layer (Fig. 1a,b; *t* = 60 min). At the same stage, an abrupt tissue fluidization of the central blastoderm occurs, with the specification zone exhibiting viscosity measurements more than an order of magnitude higher than the still pluripotent blastoderm^19^ (Fig. 1b; *t* = 30–60 min). The changes in blastoderm viscosity are the result of a rigidity phase transition restructuring the tissue in the two regions: although the pluripotent and meso-endodermal regions exhibit slight differences in control parameters of tissue rigidity, for example, cell connectivity and cell–cell adhesion strength^14,16^, they exhibit dramatic differences in tissue-scale rigidity (Extended Data Fig. 1a–d). This prompted us to hypothesize that morphogen signalling and collective tissue rigidity operate via a biochemical–biophysical feedback.

To identify a potential feedback regulation between morphogen gradients and emergent tissue properties, we combined quantitative live imaging to map the tissue material state and Nodal morphogen signalling dynamics, together with genetics and optogenetics to modulate signalling and tissue phase transitions, respectively. This allowed us to mechanistically describe a closed feedback between Nodal gradient formation and tissue phase transitions. Combining experiments with a theoretical framework that integrates tissue phase transitions and reaction–diffusion biochemical networks, we show that this feedback is functionally distinct from previously identified mechanisms where mechanics correct already established patterning via sorting^38–41^. The autoregulatory biophysical feedback identified here dynamically tunes the length scales and timescales of the Nodal gradient to adapt the exit from pluripotency to the rapidly changing tissue architecture. Therefore, this work uncovers macroscopic mechanisms of morphogen gradient formation, where transitions in collective tissue physical states do not simply define tissue deformability^14,17,19,42–46^ but directly regulate the instructive cues of cell fate decisions.

## Results

### Patterning during a rigidity transition

To explore the interplay between meso-endodermal patterning and tissue material-phase transitions, we quantified the joint spatiotemporal dynamics of cell fate and tissue material properties. As a readout of cell fate specification, we live-imaged the ventrolateral marginal blastoderm of zebrafish embryos labelled for membrane, nuclei, interstitial fluid and the meso-endodermal marker *sebox*^47^, starting at pluripotent stages (*t* = 0 min) until the onset of gastrulation (*t* = 120 min) (Fig. 1b,c,e). To quantify the material state of the blastoderm, we used rigidity percolation theory, a framework mapping the network representation of a material to its deformability^48,49^. Embryonic tissues can be abstracted as cellular networks, in which cell centroids are nodes and cell–cell contacts behave as viscoelastic bonds^14,16,50^ (Fig. 1c). Within this framework, the material response can be inferred by evaluating the size of the largest network cluster within which nodes have no independent movements, a collective property of the network referred to as the giant rigid cluster (GRC; Fig. 1c’). A transition in the GRC size occurs once the network crosses a critical point in the control parameter cell–cell connectivity, <*k*>, defined as the average number of contacts per cell^48,49,51^ (<*k*_c_> ≈ 4, approximately two-thirds of maximum average connectivity; Extended Data Fig. 1a,a’). Using this approach, we detect a transient spatial correlation between the specification zone and the rigid domain, where the former is secluded within the GRC at the blastoderm margin (Fig. 1c–e; *t* = 60–90 min). This effectively creates a physical boundary between the specifying domain and the overlaying pluripotent tissue (Fig. 1d,e, Extended Data Fig. 1e and Supplementary Movie 1). The co-occurring changes in the two macroscopic properties, meso-endodermal fate and tissue rigidity, prompted us to ask if their microscale regulators are spatiotemporally coordinated.

We explored the above question by quantifying the dynamics of the microscopic components regulating meso-endodermal fate and tissue rigidity. For meso-endodermal fate, we focused on the Nodal gradient. We performed live imaging of Smad2 and quantified the number and spatial distribution of cells with nuclear Smad2 (Fig. 1g,h, Extended Data Fig. 1f and Supplementary Movie 2). In agreement with previous reports^52–56^, we observe a short Nodal signalling range along the A–V axis, where only the first three to four most marginal cell tiers receive Nodal (closer to YSL), while cells retain the signal for ~3 h (Fig. 1h and Extended Data Fig. 1f). This pattern matches Nodal activation levels as evaluated via live imaging and quantification of the nuclear-to-cytoplasmic (N/C) ratio signal (Fig. 1i) and P-Smad2/3 immunostaining (Extended Data Fig. 1g,h), where a short-range signalling gradient is observed which steepens over time and eventually turns off.

We then quantified connectivity and GRC size along the A–V axis, to elucidate if the spatial rigidity pattern emerges as a function of spatial gradients in connectivity. We found that when the marginal tissue is still at pluripotency, the tissue is poised close to the critical point in connectivity, <*k*_c_> (Extended Data Fig. 1i). As specification progresses, connectivity increases and crosses the critical point in the most marginal cells (Extended Data Fig. 1i). The changes in connectivity seem to mainly result from a gradient in cell–cell adhesion strength (Fig. 1f,f’), which also acts as a control parameter of tissue rigidity^16,57^ (Extended Data Fig. 1b). Cell–cell adhesion strength can be inferred by the Young–Dupré relation, where the non-dimensional parameter *α*, defined as the ratio between cell–cell and cell–fluid surface tensions acting at the contact, can be derived from the angle formed at the contact edge^14,16,58–62^ (Fig. 1f and Extended Data Fig. 1b’). Quantifying *α* along the A–V axis revealed that, similarly to connectivity, at pluripotency, the tissue is poised close to the critical point of the relative surface tension (*α*_c_ ≈ 0.866) (Fig. 1f’, box 1, and Extended Data Fig. 1j) and, during specification, the most marginal cells reduce their *α* values below *α*_c_, becoming therefore more adhesive (Fig. 1f’, box 3, and Extended Data Fig. 1j). Given that the critical points in *α* and <*k*> are crossed along the A–V axis, this suggests that during the rigidity transition, the initial spatial isotropy of the tissue is broken, polarizing the GRC towards the specification zone. To test if the slight gradient in the control parameters is sufficient to polarize tissue rigidity, we simulated networks and tissues with and without a gradient in <*k*> and *α*, respectively, and explored potential changes in the GRC location along the A–V axis (Extended Data Fig. 1k–n). Networks with spatially isotropic connectivity values close to criticality position the GRC at any location along the axis (Extended Data Fig. 1k,l). By contrast, under the same <*k*> values, a slight gradient in connectivity breaks the symmetry and polarizes the GRC towards the supercritical connectivity regions (Extended Data Fig. 1k,l and Supplementary Theory Note). The same trends are seen for *α*, where in silico rigidification in the presence of a slight gradient in *α* is sufficient to polarize the GRC along the A–V axis, concentrating its presence to the subcritical regions of *α* (Extended Data Fig. 1m,n and Supplementary Theory Note).

Overall, the above results show that, during patterning, the Nodal morphogen gradient arises together with a cell–cell adhesion gradient. The latter triggers a symmetry-breaking event in the initially isotropic tissue material state, correlating the spatiotemporal dynamics of tissue rigidification to specification.

### A Nodal–tissue rigidity feedback loop

**Fig. 2:**
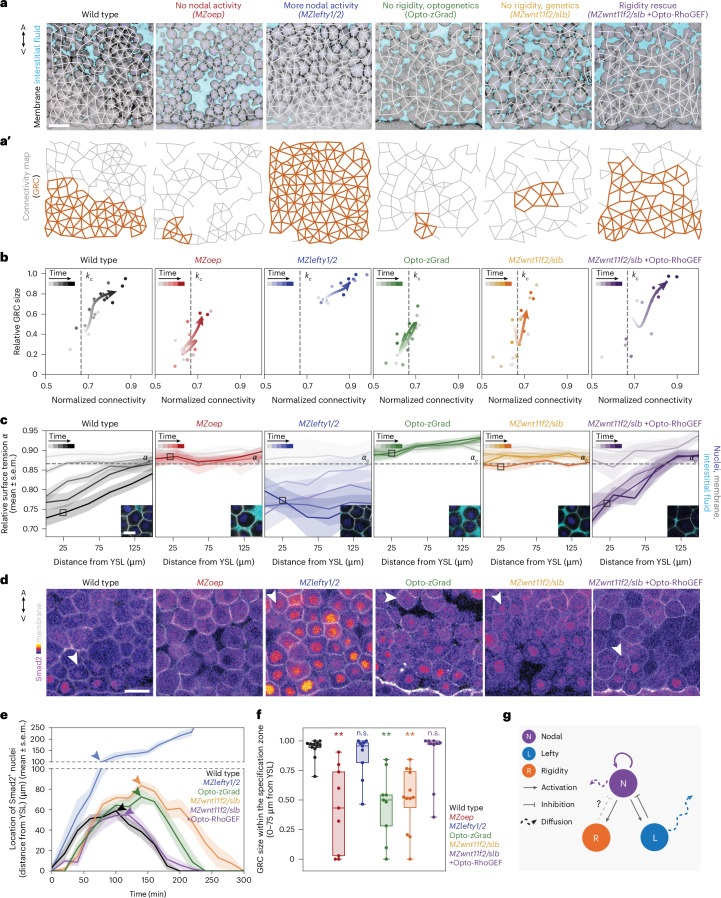
Nodal triggers a tissue rigidity phase transition along the A–V axis, which feedbacks to terminate Nodal signalling. **a**, Exemplary 2D confocal sections at *t* = 90 min for wild-type, *MZoep*, *MZlefty1/2*, Opto-zGrad, *MZwnt11f2/slb* and *MZwnt11f2/slb* +Opto-RhoGEF embryos labelled with membrane–RFP (α-catenin–citrine in Opto-zGrad) and dextran-647 for interstitial fluid (**a**), with overlaid connectivity maps and their corresponding rigidity profiles (**a’**). **b**, Plots of GRC size as a function of connectivity over time for the conditions shown in **a**. Dashed lines indicate *k*_c_. The shaded arrow connects the mean connectivity and GRC values for each timepoint (*n* = 4 embryos wild type, *n* = 3 *MZoep*, *n* = 3 *MZlefty1/2*, *n* = 3 Opto-zGrad, *n* = 4 *MZwnt11f2/slb*, *n* = 3 *MZwnt11f2/slb* +Opto-RhoGEF). **c**, Plots of the relative surface tension *α* as a function of the distance from the YSL over time for the conditions shown in **a**, with exemplary high magnification confocal sections depicting the contact angles. Dashed lines indicate *α*_c_. Data are presented as mean ± s.e.m. (wild type: *n* = 12,274 contact angles, *N* = 8 embryos; *MZoep*: *n* = 4,003, *N* = 3; *MZlefty1/2*: *n* = 3,651, *N* = 3; Opto-zGrad: *n* = 2,575, *N* = 3; *MZwnt11f2/slb*: *n* = 6,224, *N* = 6; *MZwnt11f2/slb* +Opto-RhoGEF: *n* = 2,386, *N* = 3). **d**, Exemplary 2D confocal sections of embryos from the conditions described in **a** labelled with membrane–RFP (α-catenin–citrine in Opto-zGrad) and Smad2–GFP. The white arrowheads indicate the largest distance from the YSL with Smad2-positive nuclei. **e**, Plot of the distribution of nuclear Smad2-positive cells as a function of time for the conditions shown in **a**. Arrowheads indicate the timepoints at which the images in **d** were selected, corresponding to the peak of Nodal activity length scale. Data are presented as mean ± s.e.m. (*n* = 6 embryos wild type, *n* = 3 *MZlefty1/2*, *n* = 3 Opto-zGrad, *n* = 4 *MZwnt11f2/slb*, *n* = 3 *MZwnt11f2/slb* +Opto-RhoGEF). **f**, Quantification of GRC relative size in the specification zone (0–75 μm form YSL), for all the conditions in **a**, from *t* = 60 min to *t* = 120 min. Box plots show the median (centre line), the interquartile range (IQR, box: 25th to 75th percentiles) and whiskers extending to the minimum and maximum data values (*n* = 12 wild type, *n* = 9 *MZoep*, *n* = 9 *MZlefty1/2*, *n* = 9 Opto-zGrad, *n* = 12 *MZwnt11f2/slb*, *n* = 9 *MZwnt11f2/slb* +Opto-RhoGEF) **g**, Illustration of the Nodal–Lefty–rigidity interaction network. The time axes shown in **b** and **c** correspond to time intervals of 30 min. Statistics in **f**: Kruskal–Wallis followed by Dunn’s multiple comparison test, compared with wild type. All tests were two-sided. *MZoep* *P* = 0.002. *MZlefty1/2* *P* = 0.99, Opto-zGrad *P* = 0.002, *MZwnt11f2/slb* *P* = 0.002, *MZwnt11f2/slb* +Opto-RhoGEF *P* = 0.99. Scale bars, 25 μm (**a**), 10 μm (**c**), 20 μm (**d**). n.s., non-significant. Source data

**Extended Data Fig. 2:**
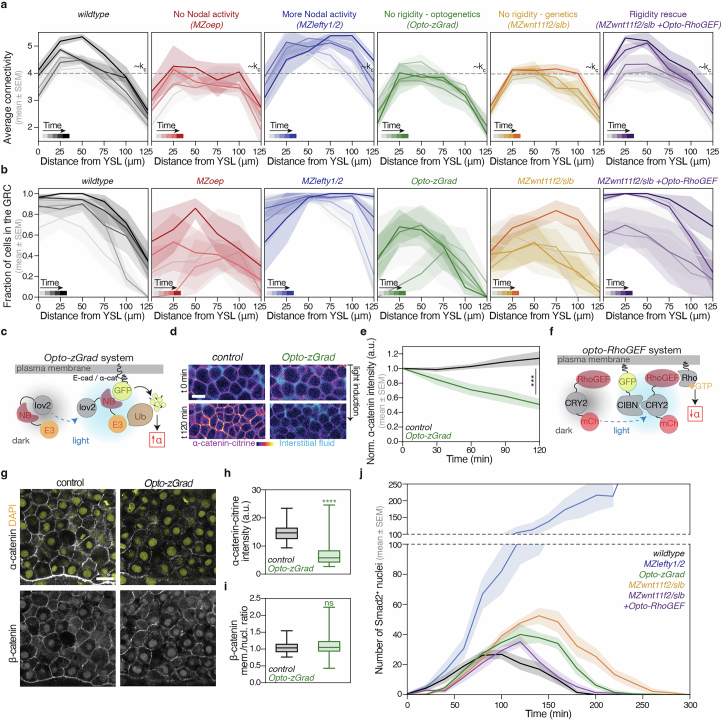
Genetic and optogenetic manipulations of Nodal signalling and tissue rigidity. Plots of average connectivity (**a**) and GRC size (**b**) as a function of the distance from the YSL over time for the experimental conditions shown in Fig. 2a. The dashed lines indicate *k*_*c*_ for large networks. Data are presented as mean ± SEM (n = 4 embryos for *wild-type*, n = 3 for *MZoep*, n = 3 for *MZlefty1/2*, n = 3 for Opto-zGrad, n = 4 for *MZwnt11f2/slb*, n = 3 for *MZwnt11f2/slb* +Opto-RhoGEF). (**c**) Schematic diagram of the Opto-zGrad system, which is composed of an AsLOV2 (LOV2) domain attached to a GFP nanobody (NB) and a domain for proteasomal degradation (E3). Upon light illumination, the GFP-NB is uncaged, so it can bind to GFP and GFP-related proteins (adapted from^16^). (**d**) Exemplary confocal images of the Opto-zGrad tool injected in a zebrafish knock-in line for α-catenin-citrine. (**e**) Quantification of the endogenous α-catenin intensity upon blue light illumination with and without Opto-zGrad, showing degradation with Opto-zGrad. Data are presented as mean ± SEM (n = 5 embryos *control*, n = 5 Opto-zGrad). (**f**) Schematic diagram of the Opto-RhoGEF tool, which combines a membrane-tagged CIBN and RhoGEF-CRY2. Upon blue light illumination, CIBN binds to CRY2, translocating RhoGEF to the membrane (adapted from^16^). (**g**) Exemplary 2D confocal sections of *wild-type* and Opto-zGrad injected embryos immunostained for DAPI and β-catenin, and quantifications of (**h**) α-catenin-citrine at the membrane and (**i**) β-catenin membrane-to-nucleus ratio fluorescent intensities. Boxplots in (h,i) show the median (centre line), the interquartile range (box: 25^th^-75^th^ percentiles), and whiskers extending to the minimum and maximum data values (n = 80 cells, N = 4 embryos each). (**j**) Plot of the number of nuclear Smad2-positive cells as a function of time for the experimental conditions shown in Fig. 2d. Data are presented as mean ± SEM (n = 6 embryos *wild-type*, n = 3 *MZlefty1/2*, n = 3 Opto-zGrad, n = 4 *MZwnt11f2/slb*, n = 3 *MZwnt11f2/slb* +OptoRhoGEF). The time axes shown in (a) and (b) correspond to time intervals of 30 min. A-V, Animal-Vegetal axis; GRC, Giant Rigid Cluster; YSL, Yolk Syncytial Layer. Statistics: (e) Unpaired t-test at t 120 min, p-value = 0.0003. (h) Unpaired t-test, p-value < 0.0001. (i) Unpaired t-test, p-value = 0.18. Scale bars: 20 μm (d, g). Source data

The correlation between the spatiotemporal dynamics of patterning and tissue rigidity prompted us to ask whether Nodal signalling and the control parameters that drive tissue rigidification are functionally linked. First, we explored if Nodal signalling drives tissue rigidification. To this end, we quantified *α*, connectivity and GRC in embryos that either lack Nodal signalling (*MZoep*, mutant for Nodal coreceptor^34,63^) or have increased Nodal signalling (*MZlefty1/2*, mutant for Nodal inhibitors^56^) (Fig. 1a’). By contrast with wild-type embryos, nuclear Smad2 signal is absent in *MZoep* embryos, confirming that Nodal signalling is impeded. Inversely, the nuclear Smad2 region expands in *MZlefty1/2* embryos, confirming that Nodal signalling is enhanced (Fig. 2a,d,e, Extended Data Fig. 2j and Supplementary Movie 2). In *MZoep* embryos, connectivity and *α* values remain close to criticality (Fig. 2b,c and Extended Data Fig. 2a), resulting in a random distribution of the GRC across the A–V axis (Fig. 2a,a’,f and Extended Data Fig. 2b). This resembles the rigidity profiles of the in silico tissues without a gradient in their control parameter (Extended Data Fig. 1k–n). Conversely, in *MZlefty1/2* mutants, connectivity and *α* values change faster (Fig. 2b,c and Extended Data Fig. 2a), with tissue rigidity percolating throughout the entire tissue (Fig. 2a,a’,f and Extended Data Fig. 2b). Altogether, these experiments show that Nodal signalling sets the spatial rigidity pattern of the zebrafish margin by regulating cell–cell adhesion and polarizing a highly connected rigid cluster of cells towards the margin (Fig. 2f,g).

As a next step, we asked if the collective material state affects meso-endoderm specification. To address this, we monitored Nodal dynamics using Smad2 live imaging in tissues with altered material properties. Our previous findings showed that tissue rigidity can be precisely altered by finetuning the relative surface tension *α* using optogenetic tools^16^. To this end, we used a degradation-based optogenetic system (Opto-zGrad)^16,64,65^ (Extended Data Fig. 2c) to maintain *α* close to *α*_c_ and, thus, inhibit the polarization of the rigid domain. Specifically, we mildly reduced α-catenin levels by inducing its degradation in a spatiotemporally resolved manner (Extended Data Fig. 2d,e) to inhibit the decrease of *α* in the most marginal cells (Fig. 2a–c,f and Extended Data Fig. 2a,b). Remarkably, when examining Nodal signalling we observed that more cells and cells further away from the YSL display nuclear Smad2, indicating a shift in the length scales of Nodal signalling in the fluidized embryos (Fig. 2d, e and Extended Data Fig. 2j). Moreover, Smad2 remains active for a longer period, suggesting that the tissue material state also influences the timescale of Nodal signalling (Fig. 2e and Extended Data Fig. 2j). Importantly, the change in Smad2 dynamics is not due to indirect effects of cell–cell adhesion on other signalling pathways, such as Wnt–β-catenin^66^, as there is no difference in β-catenin staining between wild-type and Opto-zGrad embryos (Extended Data Fig. 2g–i). Taken together, these results show that tissue rigidity can impact Nodal signalling by restricting its spatial and temporal dynamics.

Given that the adhesion gradient and emergent rigidity observed in wild-type embryos are instructed by Nodal, we hypothesized that Nodal controls its own termination not only biochemically, via the activation of its inhibitor Lefty, but also mechanically by regulating tissue rigidity. To explore such potential negative feedback, we first asked how Nodal might influence cell–cell adhesion strength. Previous work has shown that the expression of the planar cell polarity ligand, *wnt11f2*, is downstream of Nodal signalling^67^ and that *MZwnt11f2/slb* mutant embryos^68^ display weaker cell–cell adhesion and a lower blastoderm viscosity than wild-type embryos^19^. We thus analysed the dynamics of cell and tissue properties in *MZwnt11f2/slb* mutant marginal tissues over time and space. Similarly to *MZoep* embryos, the *MZwnt11f2/slb* marginal tissue exhibits no gradient in connectivity and *α* along the A–V axis (Fig. 2b,c and Extended Data Fig. 2a), which remain close to their corresponding critical points, resulting in the absence of a spatially organized rigidity pattern (Fig. 2a,a’,f and Extended Data Fig. 2b). Given that *MZwnt11f2/slb* marginal cells are competent for meso-endoderm specification^68^, we investigated the effects of the absence of polarized tissue rigidity on Nodal signalling. The fluidized mutants exhibit a higher number of nuclear Smad2-positive cells which are positioned further away from the margin, and the signal is retained longer (Fig. 2d,e, Extended Data Fig. 2j and Supplementary Movie 2), thereby phenocopying the Opto-zGrad embryos. This result supports the hypothesis that Nodal is instructing changes in tissue-scale rigidity by patterning cell–cell adhesion along the A–V axis, mediated via Wnt11f2 signalling.

If the Nodal–Wnt11f2-driven rigidity negatively feeds back to terminate Nodal signalling, one would expect to rescue the Nodal signalling dynamics observed in *MZwnt11f2/slb* mutants, solely by re-establishing tissue rigidity. To test this, we made use of the Opto-RhoGEF tool^16,69,70^ (Extended Data Fig. 2f) to increase cell contractility and reduce the relative surface tension *α* in the most marginal cells, thereby reintroducing a cell–cell adhesion gradient in the *MZwnt11f2/slb* mutants. Light activation of Opto-RhoGEF in *MZwnt11f2/slb* embryos decreases *α* in a graded manner, restoring levels comparable to those in wild-type embryos (Fig. 2c). This re-introduces a gradient in connectivity (Fig. 2b and Extended Data Fig. 2a) and polarizes tissue rigidity towards the YSL (Fig. 2a,a’,f and Extended Data Fig. 2b). Strikingly, the rescue of the tissue rigidity pattern is sufficient to fully rescue nuclear Smad2 spatial and temporal dynamics, resembling those observed in wild-type embryos (Fig. 2d,e, Extended Data Fig. 2j and Supplementary Movie 2).

Altogether, these experiments demonstrate that an adhesion-driven tissue rigidity transition negatively feeds back to Nodal signalling dynamics, in a self-generated manner.

### A rigidity transition restricts Nodal diffusivity

**Extended Data Fig. 3:**
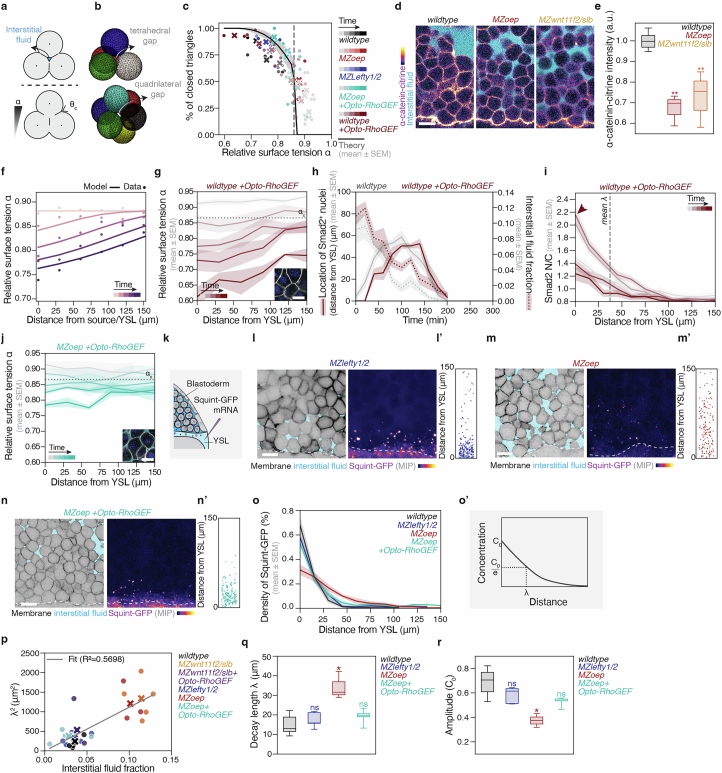
Dependency of Nodal ligand dispersal on tissue porosity. (**a**) Schematic of the closure of the interstitial fluid pocket (cyan) upon crossing of *α*_*c*_ in a 2D cell triplet, with the formation of a tricellular contact and disappearance of the interstitial fluid pocket (adapted from^16^) (**b**) Schematic of 3D cell topologies showing open tetrahedral (up) and quadrilateral (bottom) gaps. (**c**) Quantification of the percentage of closed tricellular contacts as a function of *α*. The black curve shows the simulated results of the relationship between *α* (mean ± SEM) and the closed tricellular contacts (n = 4 embryos for *wild-type*, n = 3 for *MZoep*, n = 3 for *MZlefty1/2*, n = 3 *MZoep* +Opto-RhoGEF, n = 3 for *wild-type* +Opto-RhoGEF). (**d, e**) Representative images of the endogenous α-catenin-citrine signal at t 120 min in *wild-type*, *MZoep* and *MZwnt11f2/slb* embryos at the margin (d), labelled for interstitial fluid by dextran-647 and quantifications of the endogenous α-catenin-citrine signal (e), all normalised to the *wild-type*. Boxplot shows the median (centre line), the interquartile range (box: 25^th^-75^th^ percentiles), and whiskers extending to the minimum and maximum data values (n = 7 embryos *wild-type*; n = 3 *MZoep*; n = 7 *MZwnt11f2/slb*). (**f**) Spatial profiles of *α* from simulations with feedback, overlaid with experimental values from Fig. 2c (*wild-type*). (**g**) Plot of the relative surface tension *α* as a function of the distance from the YSL over time for *wild-type* +Opto-RhoGEF with exemplary high magnification confocal sections depicting the contact angles. Dashed lines indicate *α*_*c*_. Data are presented as mean ± SEM (n = 3016 contact angles, N = 3 embryos). (**h**) Quantification of Nodal range (left y-axis, continuous line) and interstitial fluid fraction (right y-axis, dashed line) for *wild-type* (grey) and *wild-type* +Opto-RhoGEF (brown) over time. Data are presented as mean ± SEM (*wild-type*: n = 6 for Nodal range, n = 3 for porosity; *wild-type* +Opto-RhoGEF: n = 3 for Nodal range, n = 3 for porosity). (**i**) Smad2-GFP N/C ratio signal in *wild-type* +Opto-RhoGEF embryos as a function of their distance from the YSL. Timepoints are every 30 min starting from t 90 min. Dotted line at 37.22 μm indicates the mean decay length *λ* of t 90 min, t 120 min and t 150 min. Arrowhead indicates the amplitude. Data are presented as mean ± SEM (n = 3 embryos). (**j**) Same as in (g) but for *MZoep* +Opto-RhoGEF (n = 2227 contact angles, N = 3 embryos). (**k**) Schematic of the YSL injection of Squint-GFP mRNA for ligand diffusion assay. (**l-n**) (left) Exemplary 2D confocal sections of the most marginal cells in *MZlefty1/2* (l), *MZoep* (m), and *MZoep* +Opto-RhoGEF (n) embryos labelled with membrane-RFP and dextran-647 for interstitial fluid, and (right) MIPs of the same embryos labelled with squint-GFP. Dashed lines indicate YSL. (**l**’**-n**’) Plots of Squint-GFP spots distribution as a function of the distance from the YSL for the embryos in (l-n). (**o**) Plot of Squint-GFP gradient distribution for *wild-type*, *MZlefty1/2, MZoep* and *MZoep* +Opto-RhoGEF. Data are presented as mean ± SEM (n = 5 embryos *wild-type*; n = 5 *MZlefty1/*2; n = 4 *MZoep*; n = 4 *MZoep* +Opto-RhoGEF). (**o**’) Schematic diagram of the quantification of the decay length *λ* and amplitude *C*_0_. (**p**) Plot of the decay length *λ* as a function of their respective interstitial fluid fraction. The black line shows a linear fit with R^2^ indicated in the plot (numbers same as (o) and Fig. 3m) (**q, r**) Boxplots of the fitted decay length *λ* (q) and amplitude *C*_0_ (r) for each condition. Boxplots show the median (centre line), the interquartile range (box: 25^th^-75^th^ percentiles), and whiskers extending to the minimum and maximum data values (n = 5 embryos *wild-type*; n = 5 *MZlefty1/*2; n = 4 *MZoep*; n = 4 *MZoep* +Opto-RhoGEF). For (c) and (p), dots indicate single embryos and crosses indicate the mean for each condition. In (c, g, j) timepoints are every 30 min. Abbreviations: MIP, Maximum Intensity Projection; YSL, Yolk Syncytial Layer. Statistics: (e, q, r) Kruskal-Wallis test followed by Dunn’s multiple comparisons test, compared to *wild-type*: *MZoep* p-value = 0.007, *MZwnt11f2/slb* p-value = 0.008 for (e); *MZlefty1/2* p-value > 0.99, *MZoep* p-value = 0.0272, *MZoep* + Opto-RhoGEF p-value > 0.99, for (q); *MZlefty1/2* p-value > 0.99, *MZoep* p-value = 0.0109, *MZoep* + Opto-RhoGEF p-value = 0.4522 for (r). Scale bars: 10 μm (g, j), 20 μm (d, l-n). Source data

The established regulation of Nodal activity is that Nodal, as a short-range activator, induces its own expression and that of its antagonist Lefty^27,33,36,37,52^. Lefty, as a long-range repressor, inhibits Nodal activity by diffusing further away and binding to both Nodal and the coreceptors^27,52,56,71,72^. To understand how tissue rigidity regulates Nodal activity, we explored its potential involvement in the Nodal–Lefty biochemical network (Fig. 2g). Theoretical approaches indicate that the geometry of a porous environment through which particles disperse impacts diffusivity^73–75^. In the context of morphogen transport, the role of tissue architecture has been considered in poro-elastic tissues^23^, while experimental work proposed that the extracellular fluid structure can locally change morphogen concentration and diffusivity^24,28,76^. Furthermore, our recent work showed that tissue rigidification occurs in parallel to drastic changes in tissue porosity^16^. We therefore hypothesize that the rapid tissue reorganization occurring during tissue rigidification may directly influence Nodal kinetics within the timeframe of specification. The idea is underlined by the fact that, at *α*_c_, there is a sudden closure of small interstitial gaps between the cells via the formation of tricellular contacts^16^ (Extended Data Fig. 3a). This topological change leads to an abrupt collapse of the 3D interstitial fluid network (see Supplementary Theory Note Fig. SN3), in which Nodal diffuses, raising the hypothesis that a reduction in tissue porosity may restrict Nodal transport.

**Fig. 3:**
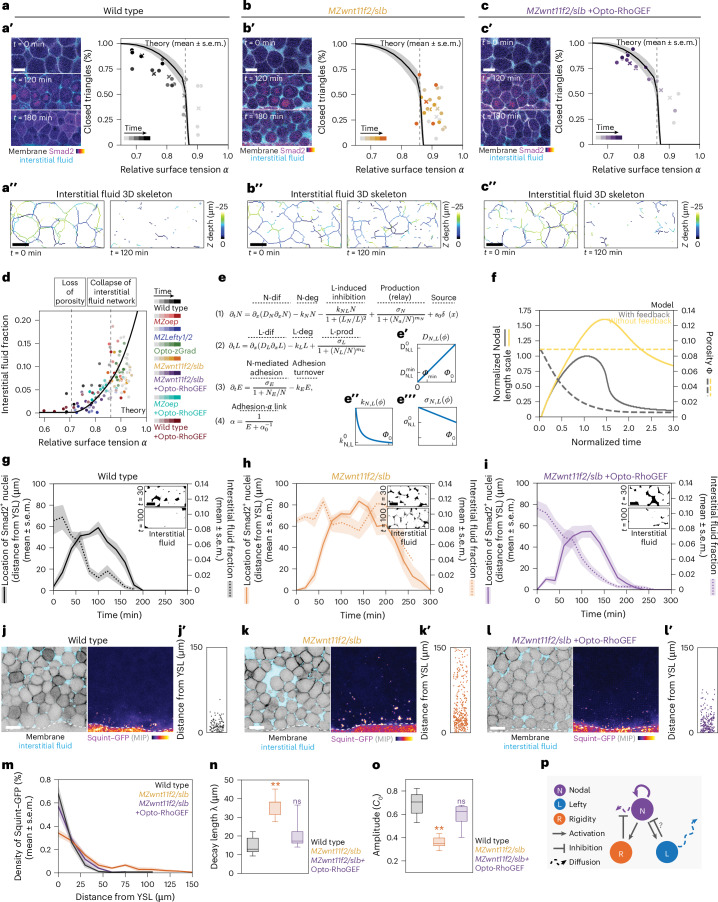
A rigidity–porosity transition negatively feeds back to Nodal signalling by restricting Nodal diffusivity. **a**–**c**, Left: Exemplary 2D confocal sections of the marginal cells close to the YSL over time in wild-type (**a’**), *MZwnt11f2/slb* (**b’**) and *MZwnt11f2/slb* +Opto-RhoGEF (**c**’) embryos labelled with membrane–RFP, Smad2–GFP and dextran-647 for interstitial fluid. Right: Quantification of the percentage of closed tricellular contacts as a function of *α* for each experimental condition. Dots indicate single embryos and crosses indicate the mean for each timepoint. Timepoints are every 30 min. Black curves show simulations of the relationship between *α* and closed tricellular contacts. Data are presented as mean ± s.e.m. (theoretical results from ref. ^16^). The dotted grey line indicates *α*_c_ (*n* = 4 embryos wild type, *n* = 4 *MZwnt11f2/slb*, *n* = 3 *MZwnt11f2/slb* +Opto-RhoGEF). **a”**–**c”**, Exemplary 3D projections of the interstitial fluid skeleton at early and late timepoints in all experimental conditions, highlighting the changes in interstitial fluid connectivity during the rigidity transition. **d**, Interstitial fluid fraction as a function of *α*, for all experimental conditions (*n* = 4 embryos wild type; *n* = 3 *MZoep*; *n* = 3 *MZlefty1/2*; *n* = 3 Opto-zGrad; *n* = 4 *MZwnt11f2/slb*; *n* = 3 *MZwnt11f2/slb* +Opto-RhoGEF; *n* = 3 *MZoep* +Opto-RhoGEF; *n* = 3 wild type +Opto-RhoGEF). Dashed lines indicate the critical points in *α* at which the tricellular gaps close and the interstitial fluid network collapses (*α*_c1_ ≈ 0.866) and all 3D multicellular gaps close and porosity is minimized (*α*_c2_ ≈ 0.707) (Supplementary Theory Note Figs. 2 and 3). **e**, Equations implemented to describe the biochemical feedback between Nodal and Lefty and between local Nodal levels and cell–cell adhesion. Equations 1 and 2 capture diffusion (dif), degradation (deg) and production (prod) of Nodal (N) and Lefty (L), respectively. The model was adapted from ref. ^77^. Equation 3 describes the accumulation of adhesion molecules as a response to Nodal levels. The plots show scaling relationships between porosity and diffusion (**e’**), degradation (**e”**) and production (**e”’**) rates (Supplementary Theory Note). **f**, Interstitial fluid fraction and Nodal range as a function of time from simulations with (grey) and without (yellow) the porosity feedback. Time is normalized to the time of maximal Nodal length scale in simulations with feedback. **g**–**i**, Quantification of Nodal range (left *y* axis, continuous line) and interstitial fluid fraction (right *y* axis, dashed line) for wild-type (**g**), *MZwnt11f2/slb* (**h**) and *MZwnt11f2/slb* +Opto-RhoGEF (**i**) embryos over time. Data are presented as mean ± s.e.m. (wild type: *n* = 6; *MZwnt11f2/slb* *n* = 4; *MZwnt11f2/slb* +Opto-RhoGEF: *n* = 3). The insets show exemplary interstitial fluid distributions at the onset and peak of Nodal signalling (*n* = 3 for all conditions). **j**–**l**, Left: Exemplary 2D confocal sections of the most marginal cells in wild-type (**j**), *MZwnt11f2/slb* (**k**) and *MZwnt11f2/slb* +Opto-RhoGEF (**l**) embryos labelled with membrane–RFP and dextran-647 for interstitial fluid. Right: MIPs of the same embryos labelled with Squint–GFP. Dashed lines indicate the YSL. **j’**–**l’**, Plots of the distribution of Squint–GFP spots as a function of the distance from the YSL for the depicted embryos in **j** to **l**. **m**, Plot of the Squint–GFP gradient distribution for the experimental conditions shown in **j** to **l**. Data are presented as mean ± s.e.m. (*n* = 5 embryos for each condition). **n**,**o**, Box plots of the fitted decay length *λ* and amplitude *C*_0_ for each condition. Box plots show the median (centre line), the IQR (box: 25th to 75th percentiles) and whiskers extending to the minimum and maximum data values (*n* = 5 embryos for each condition). **p**, Illustration of the Nodal–Lefty–rigidity network, showing the negative feedback from rigidity to Nodal effective diffusivity. Statistics for **n** and **o**: Kruskal–Wallis test followed by Dunn’s multiple comparisons test, compared with wild type. All tests were two-sided. *MZwnt11f2/slb* *P* = 0.0013 (**n**), *P* = 0.0040 (**o**); *MZwnt11f2/slb* +Opto-RhoGEF P > 0.999 (**n** and **o**). Scale bars, 20 μm (**a’**–**c’**, **a”**–**c”**, **g**–**i** and **j**–**l**). Source data

Quantification of tricellular contact formation during meso-endoderm specification revealed that, in wild-type pluripotent tissues with *α* close to *α*_c_, most of the potential tricellular contacts are open (Fig. 3a,a’). As a result, the interstitial fluid network percolates through the 3D tissue (Fig. 3a”, left). However, during specification, the tricellular contacts abruptly close when *α* crosses *α*_c_ (Fig. 3a,a’), collapsing the interstitial fluid network (Fig. 3a’’, right). The relationship between tricellular contact formation and *α* is consistent for all experimental conditions (Fig. 3a–c and Extended Data Fig. 3c), supporting the claim that the topological changes leading to the rigidity phase transition occur under the same conditions at which the geometry of the cell–cell contacts triggers the collapse of the interstitial fluid network. Quantification of tissue-scale interstitial fluid fraction as a function of *α* revealed that porosity declines even more below *α*_c_, after the tricellular contacts are closed (Fig. 3d), suggesting that porosity may be further affected by the 3D geometrical changes of multicellular contacts. We mathematically predict that although at *α*_c_ the interstitial fluid gaps vanish at tricellular points, they are still present in the central points of tetrahedral cell structures and quadrilateral gaps (Extended Data Fig. 3b and Supplementary Theory Note). By further reducing *α*, we find that at *α* ≈ 0.707 all 3D interstitial fluid gaps close in a sufficiently dense packing, reaching a minimal tissue porosity (Fig. 3d and Supplementary Theory Note). Crucially, when comparing theoretical predictions on tissue porosity against experiments, we conclude that the geometrical changes induced by the contact surface tensions can explain the changes in tissue porosity (Fig. 3d). The dynamic but drastic changes in tissue architecture occurring during specification suggest that concomitant effects on Nodal transport may arise.

To investigate how the spatial and temporal dynamics of Nodal could be altered by the dynamic changes in tissue porosity, we developed a theoretical framework that integrates a biochemical feedback between Nodal and Lefty on the basis of previous reports^27,52,77^, together with a feedback between ligand dispersal and tissue porosity (Fig. 3e). We first obtained scaling relationships between tissue porosity and morphogen diffusion, degradation and production from first principles (Fig. 3e’–e”’ and Supplementary Theory Note). To model the response of tissue porosity to Nodal levels, we assumed that *α* is a function of adhesion^16,58^ and that Nodal activates adhesion factors. These assumptions were supported by our experimental data showing that tissues undergoing rigidification also display increased levels of α-catenin, which are Nodal/Wnt11f2 dependent (Fig. 3e, equations 3 and 4, and Extended Data Fig. 3d–f). We further used the derived relationship between tissue porosity and *α* (Fig. 3d and Supplementary Theory Note). On the basis of these assumptions, we found that, in the presence of feedback between Nodal and porosity, the coordinated dynamics of Nodal and porosity are consistent with those seen in wild-type embryos: Smad2 turns on and reaches its peak, followed by a sharp drop in porosity and, then, a timely termination of the Smad2 signal (Fig. 3f, grey lines, and Fig. 3g). In the absence of feedback between Nodal and porosity, the model predicts that, while porosity remains constant, Nodal signalling reaches further away from the source and takes longer to switch off (Fig. 3f, yellow lines). Importantly, this profile matches the relationship between porosity and Nodal dynamics in the *MZwnt11f2/slb* embryos, which display little decline of the interstitial fluid fraction, showing a larger range of Smad2 and a delay in turning off (Fig. 3h). Experimentally, the spatiotemporal Nodal dynamics in *MZwnt11f2/slb* are fully rescuable when restoring tissue rigidity and porosity dynamics via optogenetic increase of cell–cell adhesion strength (Fig. 3i). Further increasing cell–cell adhesion strength using optogenetics in already rigid tissues (wild type+Opto-RhoGEF) (Extended Data Fig. 3g) does not further reduce tissue porosity because porosity plateaus beyond a certain value of relative surface tension *α* (Fig. 3d and Extended Data Fig. 3h). Importantly, these tissues display Nodal levels of similar length and timescales (Extended Data Fig. 3h,i), suggesting that the restriction of the Nodal spatiotemporal range is unlikely to reflect a mechanosensitive crosstalk between cell contractility and cell–cell adhesion with Nodal signalling^78^. Instead, the rigidity transition and the concomitant reduction in tissue porosity appear as the main physical factors restricting Nodal dynamics.

Given that the model assumes that tissue porosity changes Nodal diffusivity, we experimentally assessed the effects of porosity on Nodal dispersal. To test this, we expressed a fluorescently-tagged version of the Nodal ligand Squint in the YSL^27,72^ (Extended Data Fig. 3k) and quantified its length scale. In wild-type embryos, which rigidify and have reduced porosity, the Nodal ligand gradient is short range, in agreement with previous reports^27,52,79^ (Fig. 3j,j’). However, in *MZwnt11f2/slb* embryos, where the tissue is more porous, the Nodal ligand becomes long range (Fig. 3k,k’). This phenotype could be fully rescued by optogenetically inducing rigidification in the mutant, reverting Squint to a short-range ligand (Fig. 3l,l’). Given that Nodal diffusivity is also expected to be regulated by Nodal interactions with its receptors and coreceptors and Nodal secretion by cells^27,71^, we performed a series of experiments to explore Nodal ligand dispersal in embryos where Nodal secretion or receptors are impaired, while optogenetically manipulating tissue porosity. To this end, we quantified ligand dispersal across three conditions: *MZlefty1/2* embryos, displaying increased Nodal secretion and low porosity (Fig. 3d and Extended Data Fig. 3l,l’); *MZoep* embryos, which lack ligand binding, uptake and secretion and exhibit high porosity (Fig. 3d and Extended Data Fig. 3m,m’); and *MZoep* +Opto-RhoGEF embryos, which similarly lack ligand binding, uptake and secretion but are mechanically rigid with low porosity (Fig. 3d and Extended Data Fig. 3j,n,n’). A systematic analysis of the spatial profile of the Nodal ligand across all of the above experimental conditions revealed an exponential-like decay (Fig. 3m and Extended Data Fig. 3o). When we quantified the decay length *λ* and amplitude *C*_0_ (Fig. 3n,o and Extended Data Fig. 3o’,q,r), we could show that *λ* changes as a function of porosity (Extended Data Fig. 3p) and that more porous and fluid-like embryos (*MZwnt11f2/slb*, *MZoep*) display increased *λ* and decreased *C*_0_ when compared with less porous and solid-like embryos (wild type, *MZwnt11f2/slb* +Opto-RhoGEF*, MZoep* +Opto-RhoGEF*, MZlefty1/2*) (Fig. 3n,o and Extended Data Fig. 3q,r), including conditions where Nodal binding, uptake and secretion properties are impaired. Given that an increased *λ* together with decreased *C*_0_ is a signature of increased diffusivity (Supplementary Theory Note Fig. SN8), the above changes in the Nodal ligand profile strongly suggest that tissue rigidification primarily physically restricts Nodal effective diffusivity.

### A tissue rigidity transition is timing Nodal–Lefty dynamics

**Fig. 4:**
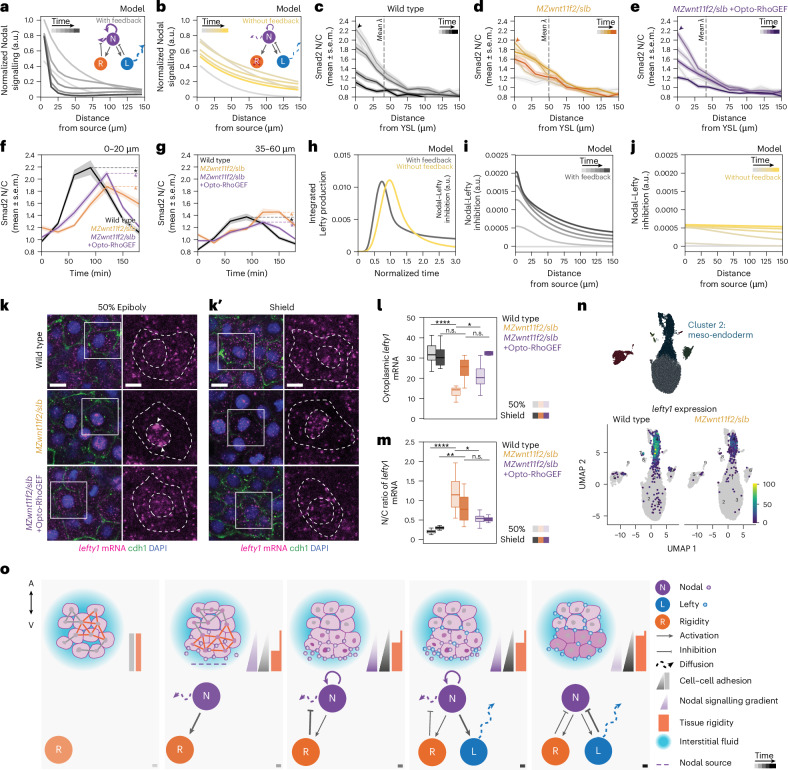
A rigidity transition enhances the Nodal–Lefty biochemical network. **a**,**b**, Simulations of Nodal signalling activity in conditions with porosity feedback (**a**) and without (**b**) over space and time. Timepoints are from 0.25 to 2.75 of normalized time, as shown in Fig. 3f. **c**–**e**, Smad2–GFP N/C ratio signal in wild-type (**c**), *MZwnt11f2/slb* (**d**) and *MZwnt11f2/slb* +Opto-RhoGEF (**e**) embryos as a function of their distance from the YSL. Timepoints are every 30 min starting from *t* = 90 min. Dotted lines indicate the mean decay length *λ* of *t* = 90 min, *t* = 120 min and *t* = 150 min: wild type, 40.8 μm, *MZwnt11f2/slb*, 49.7 μm, *MZwnt11f2/slb* +Opto-RhoGEF, 41.2 μm. Data are presented as mean ± s.e.m. (*n* = 3 embryos for each condition). Arrowheads indicate the amplitude. **f**,**g**, Quantification of Smad2–GFP N/C ratio signal for the conditions shown in **c** to **e** at the cells close to (0–20 μm) (**f**) and further away from the YSL (35–60 μm) (**g**) over time. Asterisks indicate Nodal maximum levels. Data are presented as mean ± s.e.m. (*n* = 3 embryos for each condition). **h**–**j**, Simulations of the integrated Lefty production over time (**h**) and the Nodal–Lefty inhibition over space and time (**i** and **j**) with and without the porosity feedback. Timepoints are from 0.25 to 2.75 of normalized time, as shown in Fig. 3f. **k**,**k’**, Exemplary images of *lefty1* mRNA labelled via smFISH of wild-type, *MZwnt11f2/slb* and *MZwnt11f2/slb* +Opto-RhoGEF embryos at 50% epiboly (**k**) and shield (**k’**) stages. Left: *lefty1* mRNA in magenta, cell membranes labelled with anti-E-cadherin (cdh1) antibody in green and nuclei with DAPI in blue. Right: zoomed-in images of the cells indicated in the white box, with *lefty1* mRNA in magenta and the white dashed lines marking membrane and nuclear boundaries. White arrowheads indicate transcriptional hubs. **l**,**m**, Plots of the amount of cytoplasmic *lefty1* mRNA spots per cell on the mid-2D plane (**l**) and N/C ratio of the *lefty1* mRNA spots per cell (**m**) in wild-type, *MZwnt11f2/slb* and *MZwnt11f2/slb* +Opto-RhoGEF embryos at 50% epiboly (dim-shaded boxes) and shield (dark-shaded boxes) stages. Box plots show the median (centre line), the IQR (box: 25th to 75th percentiles) and whiskers extending to the most extreme values within 1.5 × IQR (wild type 50%, *n* = 14 embryos, shield, *n* = 7; *MZwnt11f2/slb* 50%, *n* = 13, shield *n* = 6; *MZwnt11f2/slb* +Opto-RhoGEF 50%, *n* = 14, shield *n* = 6). Minimum ten cells per embryo. **n**, Top: Uniform Manifold Approximation and Projection (UMAP) of cells derived from wild-type and *MZwnt11f2/slb* embryos at shield stage profiled with single-cell sequencing, coloured by cluster. Clusters were defined in UMAP space, and cluster 2 in blue indicates the meso-endoderm. Bottom: *lefty1* expression in wild-type and *MZwnt11f2/slb* embryos on UMAP from single-cell RNA sequencing data. **o**, Schematics illustrating a closed feedback between Nodal signalling and tissue rigidity. Statistics in **l** and **m**: Kruskal–Wallis test followed by Dunn’s multiple comparisons test, *MZwnt11f2/slb* compared with wild type at 50% epiboly *P* < 0.0001 and at shield *P* = 0.1874 for **l**, 50% epiboly *P* < 0.0001 and shield *P* = 0.0041 for **m**, *MZwnt11f2/slb* +Opto-RhoGEF compared with *MZwnt11f2/slb* at 50% epiboly *P* = 0.0148 and at shield *P* = 0.0532 for **l**, 50% epiboly *P* = 0.0199 and shield *P* > 0.9999 for **m**. Scale bars, 10 μm (left), 5 μm (right) in **k** and **k’**. Source data

**Extended Data Fig. 4:**
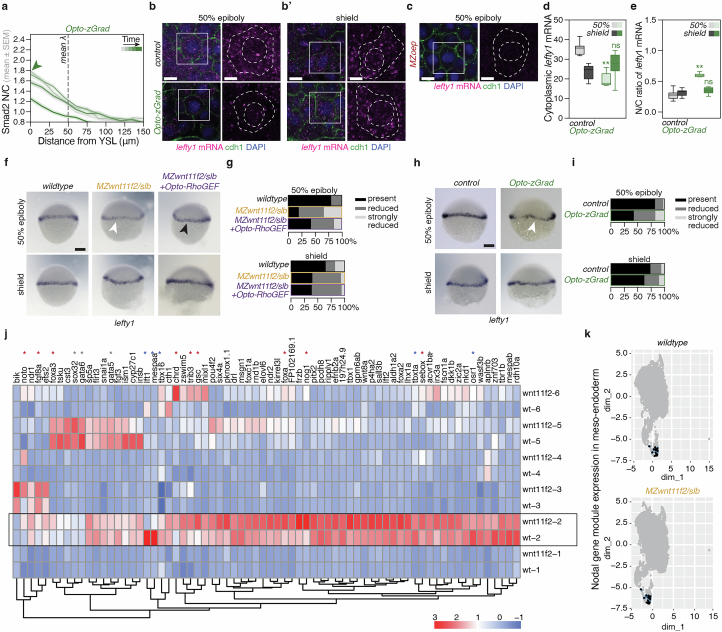
Gene expression analysis with and without the rigidity transition. (**a**) Smad2-GFP N/C ratio signal in Opto-zGrad embryos as a function of their distance from the YSL. Timepoints are every 30 min starting from t 90 min. Dotted line at 49.88 μm indicates the mean decay length *λ* of t 90 min, t 120 min and t 150 min. Arrowhead indicates the amplitude. Data are presented as mean ± SEM (n = 3 embryos). (**b, c**) Exemplary images of *lefty1* mRNA labelled via smFISH of *control*, Opto-zGrad and *MZoep* (**c**) embryos at 50% epiboly (**b**, **c**) and shield (**b**’) stages. Left panel: marginal tissue overview with *lefty1* mRNA in magenta, cell membranes labelled with anti-E-Cadherin (cdh1) antibody in green and nuclei with DAPI in blue. Right panel: zoom in of a cell with *lefty1* mRNA in magenta, white dotted lines mark membrane and nuclear boundaries. (**d, e**) Plots of the amount of cytoplasmic *lefty1* mRNA spots per cell on the mid-2D plane (d) and nuclear to cytoplasmatic ratio of the *lefty1* mRNA spots per cell (**e**) in *control* and Opto-zGrad embryos at 50% epiboly stage (dim-shaded box) and shield stage (dark-shaded box). Box plots show the median (centre line), the interquartile range (box: 25^th^-75^th^ percentiles), and whiskers extending to the most extreme values within 1.5xIQR (*control* 50%: n = 7 embryos, shield: n = 6; Opto-zGrad 50%: n = 6, shield n = 6. Minimum 10 cells per embryo). (**f**) Representative brightfield images of *lefty1* expression visualized via *in situ* hybridisation in *wild-type*, *MZwnt11f2/slb* and *MZwnt11f2/slb* +OptoRhoGEF at 50% epiboly and shield stage. White arrowhead indicates downregulation and black arrowhead sufficient expression. (**g**) Percentage of embryos showing *lefty1* expression, reduced expression, strongly reduced expression at 50% epiboly (top) and shield (bottom) stages (*wild-type* 50%: n = 10 embryos, shield: n = 17; *MZwnt11f2/slb* 50%: n = 15, shield n = 15; *MZwnt11f2/slb* +OptoRhoGEF 50%: n = 14, shield: n = 18) (**h**) Representative brightfield images of *lefty1* expression visualized via *in situ* hybridisation in *control* and Opto-zGrad at 50% epiboly and shield stage. (**i**) Percentage of embryos showing *lefty1* expression, reduced expression, strongly reduced expression at 50% epiboly stage (top) and shield stage (bottom) (*control* 50%: n = 39 embryos, shield: n = 49; Opto-zGrad 50%: n = 40, shield n = 40). (**j**) Expression levels from single-cell RNA sequencing data for a set of Nodal target genes across all clusters in *wild-type* and *MZwnt11f2/slb* embryos, with most genotype-specific differences concentrated in meso-endoderm (box, cluster 2). Red asterisks indicate upregulated genes, blue asterisks indicate downregulated genes and grey asterisks indicate unchanged gene expression. (**k**) Gene module UMAP, where each point represents a single gene and genes are grouped based on their co-expression in meso-endoderm cells. Known Nodal target genes (highlighted in blue) co-localize in the gene module UMAP, reflecting a shared co-expression pattern that is intact in both *wild-type* (left) and *MZwnt11f2/slb* (right) shield-stage embryos. N/C, Nuclear-to-cytoplasmic ratio. Statistics: unpaired t-test, 50% epiboly p-value = 0.0012, shield p-value = 0.3095 (**d**) and 50% epiboly p-value = 0.0023, shield p-value = 0.4848 (e). Scale bars: 10 μm (left) and 5 μm (right) (b, b’, c), 200 μm (**f**, **h**). Source data

Although the shift in the Nodal ligand gradient can explain how rigidity restricts the length scale of Nodal signalling, it is yet unclear how it restricts its timescale. To this end, we asked whether the porosity-dependent changes in the Nodal length scale may, in turn, modify the temporal dynamics by which Nodal transcriptionally activates its downstream targets^80^, including its inhibitor Lefty (Fig. 3p). To investigate this idea, we returned to the theoretical model, and we first evaluated the shape of the Nodal signalling gradient, which defines the transcriptional response, with and without feedback. Assuming that both tissues of low and high porosity receive the same influx of Nodal from the YSL, the model predicts that in the presence of feedback, Nodal activity should form a sharper signalling gradient with higher amplitude at the source (Fig. 4a). By contrast, in the absence of feedback, at the same timescales, Nodal activity should form a shallower signalling gradient with a lower amplitude (Fig. 4b). To test the predictions of the model, we quantified Nodal activity in wild-type, *MZwnt11f2/slb, MZwnt11f2/slb* +Opto-RhoGEF and optogenetically fluidized Opto-zGrad embryos by measuring the Smad2 N/C ratio (Fig. 4c–e and Extended Data Fig. 4a). Accordingly, the cells closer to the YSL in wild-type and *MZwnt11f2/slb* +Opto-RhoGEF embryos displayed a higher peak in Nodal activity, compared with *MZwnt11f2/slb* fluidized and optogenetically fluidized Opto-zGrad embryos (Fig. 4c–e, arrowheads, Fig. 4f, asterisks, and Extended Data Fig. 4a). In addition, cells further away from the YSL had higher Nodal activity in the fluidized embryos (Fig. 4g, asterisks), forming a shallower activity gradient (larger *λ*; Fig. 4c–e and Extended Data Fig. 4a, dashed line). These findings show that the effects of the rigidity on the ligand gradient directly impact the amount of Nodal that cells receive. A logical consequence is that the transcriptional response downstream of Nodal may differ between rigid and fluid tissues.

Given that a crucial downstream target of Nodal is its inhibitor Lefty, which is important for terminating Nodal signalling, we theoretically explored the dynamics of Lefty production when tissue porosity is, or is not, Nodal dependent. We observe that in the presence of feedback, where Nodal activity is enhanced near the source, *lefty* production starts earlier than in the absence of feedback (Fig. 4h). This could in principle accelerate the termination of Nodal signalling because a source of Nodal clearance is Lefty^27,36,37,56^. To test this, we quantified the strength of the inhibition of Nodal by Lefty in the model which is predicted to be higher and faster in the presence than in the absence of feedback (Fig. 4i,j). This prediction suggests that tissue rigidity effectively amplifies and expedites the Lefty–Nodal negative biochemical feedback. To test the model predictions, we performed single-molecule fluorescent in situ hybridization (smFISH) and ISH for *lefty1* RNA in wild-type, *MZwnt11f2/slb*, *MZwnt11f2/slb* +Opto-RhoGEF and Opto-zGrad embryos during specification (Fig. 4k–m and Extended Data Fig. 4b–i). This revealed that in rigid tissues of low porosity, *lefty1* is sufficiently expressed early during specification, with most transcripts localized in the cytoplasm (Fig. 4k and Extended Data Fig. 4f,g). By contrast, in fluidized and highly porous earlier stage embryos, *lefty1* expression is overall lower, the transcriptional hubs are still active and most of the transcripts are located in the nucleus (Fig. 4k–m, arrowheads, and Extended Data Fig. 4b–i), and this phenotype starts to revert at later stages (Fig. 4k’–m and Extended Data Fig. 4b’,d–i). The delay in *lefty1* expression is not due to a generic developmental delay in the mutant embryos because optogenetic rescue of tissue rigidity partially restores the early expression of the antagonist, also reflecting the *lefty1* transcription dynamics of Opto-zGrad embryos (Fig. 4k–m and Extended Data Fig. 4b–i). These results indicate that tissue rigidification regulates the timing of *lefty1* expression and, consequently, the strength of the Nodal–Lefty negative biochemical feedback. In this way, Nodal enhances its own degradation biochemically, by inducing its own inhibitor and, mechanically, by reducing its diffusivity and speeding up the induction of its inhibitor.

Self-enhanced morphogen negative regulation was previously shown to endow robustness to morphogen gradients^81,82^, raising the hypothesis that the before-described double-negative feedback may be important for robust gene expression. To address the downstream consequences of uncoupling the Nodal–Lefty biochemical network dynamics from the tissue rigidity transition, we performed single-cell RNA sequencing in wild-type versus *MZwnt11f2/slb* mutant embryos (Fig. 4n, top). We identified the Nodal module using two approaches. First, we identified genes whose expression correlated with a curated list of Nodal targets in meso-endoderm cells^83^ and examined expression levels of genes in this module across cell types (Extended Data Fig. 4j). Second, we performed unsupervised co-expression analysis of all expressed genes in meso-endoderm by reducing dimensionality of transposed cell × gene matrix to cluster genes into modules^84^. In this gene embedding space, we found a cluster of co-expressed genes that contained all curated Nodal targets, and this module was intact in both wild-type and *MZwnt11f2/slb* embryos (Extended Data Fig. 4k). This dataset confirmed that *lefty1* expression is downregulated in the fluidized *MZwnt11f2/slb* embryos (Fig. 4n, bottom) but also several Nodal targets are upregulated, including *fgf8a*, *gsc*, *chrd* and *noto* (Extended Data Fig. 4j, red asterisks). Notably, several Nodal targets were unaffected (*gata5* and *sox32*; Extended Data Fig. 4j, grey asterisks) or even downregulated (*tbxta* and *tbx16*; Extended Data Fig. 4j, blue asterisks), suggesting that the meso-endodermal patterning appears fragile in *MZwnt11f2/slb* embryos, reminiscent of the phenotype of *MZlefty1/2* mutants^56^. These findings are consistent with the change in the shape of the Nodal signalling gradient in the absence of the rigidity feedback. Shallower gradients with smaller amplitudes imply that cells closer to the source may not receive sufficiently high levels of Nodal to activate genes that are transcribed at high Nodal thresholds (Fig. 4c,d, arrows, Fig. 4f, asterisks). By contrast, because Nodal decays slower in fluidized embryos, cells further away from the source experience higher levels of Nodal than in the rigid embryos, resulting in the overtranscription of genes that require low levels of Nodal (Fig. 4g, asterisks). Therefore, the changes in the Nodal signalling gradient induced by the rigidity transition are directly impacting gene expression, distorting positional information. Together, these results propose the negative feedback from emergent tissue properties as a mechanism for timing the Nodal–Lefty reaction–diffusion dynamics to ensure precise patterning of the meso-endodermal domain (Fig. 4o).

## Discussion

By uncovering the mechanisms coupling the nonlinear dynamics of a tissue rigidity transition to the Nodal–Lefty reaction–diffusion dynamics, this work demonstrates that collective tissue properties shape morphogen signalling gradients. The regulation of the timing of cell signalling dynamics by the emergent tissue mechanics unveils a novel mechanism for coordinating a position-appropriate gene expression programme to the concomitant tissue structural changes.

Nodal signalling is a paradigm for morphogen-mediated patterning because it displays signatures of wide-spread mechanisms of morphogen gradient formation, including hindered diffusion, the action of signalling feedback and relay, and receptor sensitivity and binding^27,52,54,71,72,85^. In the context of hindered diffusion, the Nodal signalling range has been shown to be restricted by its binding to the co-receptor *oep*^71^. Similarly, we find that in *MZoep* mutant embryos the Nodal effective diffusivity increases, consistent also with an increase in tissue porosity. Notably, reducing tissue porosity in *MZoep* mutants still had a major effect on Nodal ligand dispersal. These results suggest that drastic and abrupt changes in tissue architecture mediated by phase transitions can govern large-scale distribution of ligands within tissues, presumably directing ligands towards their receptors and thereby facilitating binding. At the critical point of the rigidity transition, the cell–fluid interfaces vanish owing to the closure of the tricellular contacts, inducing the collapse of the interstitial network. At the cell–cell interfaces, Nodal was shown to have slower diffusion but a higher bound fraction^76^, suggesting that a sharp drop in porosity may enhance Nodal binding. This could further enhance downstream signalling activity by facilitating local production and Nodal relay to the immediate neighbours^52,79,86^ to shape the gradient. Accordingly, our numerical simulations capture the Nodal–Nodal and Nodal–Lefty interactions and the impact of porosity on this biochemical network when varying the relative contribution to Nodal dispersal via relay (Supplementary Theory Note Figs. SN6 and SN7). This suggests that porosity can restrict local diffusivity and have a similar impact on Nodal dynamics even when transport is not solely based on diffusion.

In general, tissue porosity and the network topology of the extracellular compartments emerge as key regulators of morphogen transport and patterning across developing systems^87^. Recent work showed that on the one hand, the topology of the interstitial fluid network can impede morphogen transport, as in the case of Hedgehog proteins^25^, but on the other hand, facilitate FGF transport^24^ or even concentrate it in extracellular compartments^28^. As biophysical properties of morphogens and of the extracellular space probably define how morphogens are transported (for example, diffusion- versus cell based), porosity changes could broadly affect not only the range of morphogen activity but also additional functionalities such as scaling or the interpretation of combined morphogen signalling^77,88–92^. Moreover, our finding that porosity is nonlinearly regulated via material-phase transitions can provide simple solutions for limitations of the hindered diffusion-based models. This would be the case when physical boundaries are absent and a morphogen could leak out of the tissue of interest^93,94^. We show that when the tissue undergoes a rigidity transition, a material boundary effectively appears at the same time. The emergence of such a solid-like highly connected cellular boundary could act as a barrier that impedes morphogen dispersal within the interstitial fluid. Given that the material boundary is also secluding the specifying cells, it may further offer mechanical isolation of the specification zone from neighbouring tissues facilitating germ layer segregation^41,95^.

Finally, diffusive molecules, such as morphogens, may be ideal physiological regulators of control parameters for tissue phase transitions—such as cell adhesion, motility, shape or connectivity—because they can generate graded changes in cell states which, in turn, may induce highly nonlinear responses in the macroscopic tissue state. Our findings show that when morphogen gradients directly regulate cell control parameters, they trigger structural symmetry-breaking events in space and rapid transitions in tissue organization in time. The above multiscale regulation could serve as potential generic mechanism by which morphogen-induced changes in supracellular mechanics feedback to morphogen gradients^96^. For instance, an initiation or arrest of cell motility or growth in epithelia could trigger tissue fluidization or solidification, which could in turn facilitate or hinder the dispersal of cell-bound morphogens, respectively^20,97–99^. Therefore, the closed spatiotemporal feedback between tissue organization and morphogen gradients proposes a conceptual framework of how patterning and tissue-scale physical properties interact: morphogen-driven emergent tissue properties act as a design principle that allows morphogens to quickly adjust their own length scales, ensuring that cell fate specification is kept in sync with the rapidly changing tissue architecture.

## Methods

### Zebrafish handling

All animal experiments were carried out according to the guidelines of the Committee for Animal Welfare and Institutional Animal Care and Use (IACUC) under the European Molecular Biology Laboratory (EMBL) Policy on the Protection and Welfare of Animals Used for Scientific purposes (IACUC code 21-010_HD_NP).

Zebrafish (*Danio rerio*) were raised at 28.5 °C under a 14 h light–10 h dark cycle^100^. The following zebrafish strains were used in this study: wild type A2B2, Tg(mezzo:eGFP)^101^, *MZwnt11f2/slb*^tx226^ (ref. ^68^), *MZoep*^tz257^ (ref. ^102^), *MZlefty1*^a145^*lefty2*^a146^ (ref. ^56^) and *Gt(ctnna-citrine)*^*ct3a*^ (ref. ^103^). From the above lines, this study generated the following lines: *MZwnt11f2/slb*^tx226^;Tg(mezzo:eGFP), *MZwnt11f2/slb*^tx226^;*Gt(ctnna-citrine)*^ct3a^ and *MZoep*^tz257^;*Gt(ctnna-citrine)*^ct3a^. For raising, *MZoep*^tz257^ embryos were rescued with injection of 200 pg *oep* mRNA and *MZlefty1/2* mutants were rescued by growing the embryos in 2.5 µM Nodal inhibitor in E3 from single-cell stage for 24 h (S4696 Sigma-Aldrich, SB505124). Zebrafish embryos were kept at 28.5 °C and maintained in 1× Danieau’s medium for all experimental incubations. Staging was performed as previously described^104^.

### Embryo microinjections

Zebrafish embryos were injected using glass capillary needles (30-0020, Harvard Apparatus) that were pulled with a P-97 needle puller (Sutter Instrument) and attached to a PV820 microinjector system (World Precision Instruments). mRNA in vitro transcription was performed with the mMessage mMachine SP6 kit (Thermo Fisher Scientific, AM1340). The following amounts were injected at the single-cell stage in the yolk: 70 pg membrane–RFP^105^, 20 pg histone–BFP^106^, 50 pg Smad2–GFP^107^, 35 pg Opto-Zgrad^16^, 70 pg CIBN-CAAX^16^ and 15 pg Opto-RhoGEF2–CRY2^16^. Interstitial fluid was labelled with 0.5 nl 0.6 μg μl^−1^ 10,000 MW Dextran-Alexa 647 (Thermo Fisher Scientific, D22914) injection in the blastoderm at 1K-oblong stages (3–3.5 hours post fertilization). Ligand YSL injections were performed at sphere stage by co-injecting in the YSL 30 pg histone–BFP mRNA, 170 pg squint–GFP mRNA^27^ and 0.68% phenol red (Sigma, P0290). Phenol red was used to evaluate the success of the injection.

### In situ hybridization

In situ hybridizations were performed as previously described^108^. In brief, 50% epiboly-stage and shield-stage embryos were fixed with 4% paraformaldehyde (PFA; EMS, E15713) in PBS overnight at 4 °C, then washed in PBS, dechorionated and stored in 100% methanol at −20 °C. Embryos were then rehydrated in PBS, permeabilized in PBS + Tween 20 0.1% (PBT) and incubated in hybridization buffer (50% Formamide, 5X SSC, 0.1% Tween 20, 50 μg ml^−1^ heparin, 500 μg ml^−1^ transfer (t)RNA and citric acid) with digoxigenin-labelled RNA probe for *lefty1* overnight (Heisenberg laboratory; *lefty1* RNA probe was synthesized with the DIG RNA labelling Kit (Roche, 11175025910)). Embryos were washed in serial dilutions of SSC, then incubated overnight with an alkaline phosphatase antidigoxigenin antibody (11093274910, Roche), washed and stained with NBT (11383213001, Roche) and BCIP (11383221001, Roche) and the reaction was stopped with 100% ethanol. Subsequently, samples were stored in PBS.

### Immunostaining

Embryos at needed stages were fixed in 4% PFA solution in PBS overnight at 4 °C. The next day, samples were washed 3 × 5 min in PBS, manually dechorionated and then stored in 100% methanol at −20 °C. Embryos were rehydrated in PBS and washed 5 × 5 min in PBS + 1% TritonX-100 (PBSTr), blocked 2 × 2 h in blocking solution (PBSTr + 10% goat serum + 1% dimethyl sulfoxide) and incubated overnight at 4 °C with primary antibody anti-P-Smad2/3 (8828S, Cell Signalling, MAb, Clone D27F4, Lot 8) at 1:1,000 in blocking solution, as described in ref. ^53^. Samples were washed 5 × 10 min in PBSTr, 3 × 5 min in PBS + 0.1% TritonX-100 (PBTr) for 1 h and incubated overnight at 4 °C with secondary antibody (1:500, antirabbit Alexa 647, Invitrogen, A21244, Lot 2247991). Samples were then washed 2 × 5 min in PBTr, labelled with 4,6-diamidino-2-phenylindole (DAPI; 300 nM, D1306, Invitrogen) for 20 min and washed 5 × 10 min in PBSTr. Embryos were transferred to 80% glycerol in PBS with 0.4% *N*-propyl-gallate. β-catenin immunostainings were performed as described above, with the difference that embryos were not stored in methanol, were blocked in blocking solution (PBS + 1% Triton + 2% BSA for 3 h) and anti-β-catenin primary antibody incubation (C7207, Sigma-Aldrich, MAb, Clone 15B8, Lot 089M4857V) was performed overnight at 4 °C (1:100 in PBS + 0.5% Triton + 2% BSA). The next day, the samples were washed 5 × 10 min with PBSTr (PBS + 0.5% Triton). Secondary antibody (antimouse Alexa 647, Invitrogen, A21235, Lot 2272554) incubation was performed at room temperature for 4 h (1:500 in PBS + 0.5% Triton + 2% BSA). Samples were stained with 300 nM DAPI for 20 min in PBS + Tween 20 0.1% (PBSTw), then washed 2 × 5 min in PBSTw and then transferred to PBS for imaging.

### smFISH

Single-molecule fluorescence in situ hybridization (smFISH) was performed by adapting the SABER-FISH protocol^109^ and omitting the signal amplification by exchange reaction. Probe design for the zebrafish *lefty1* coding sequence (ENSDART00000019196.7) was performed on paintshop.io^110^: 25 probes of 29–36 base pairs (bp) were designed and checked for cross-reactivity with ortholog genes. *lefty1* probes are listed here:

lefty1-pr-1: AACTCCTCTAGGTTGAGTGTGTAAAGCACCAGCT,

lefty1-pr-2: GCGTAACTGCCGGGTCTCTCGCCCTCGATC,

lefty1-pr-3: CACACCTCAAGGTGCATGGGCATCTCCATT,

lefty1-pr-4: CTGGTTTTCCCAGAGTGTTGTCGTCTGGGTC,

lefty1-pr-5: TTGAGTTGTGAAGTGGACACACTTGGCCATCTCT,

lefty1-pr-6: GTCTTTCTGAGGTTCCACCCAGTAGATGCTCACT,

lefty1-pr-7: CGTGCGTTGTTGACCGGTCTGTGGCCCTTT,

lefty1-pr-8: CTCTCCGGTATGGAGCGCTTGTGTGGGGCC,

lefty1-pr-9: TTCTTGTAGAGCTTCAGTTCTGCCATGGTCACTTCA,

lefty1-pr-10: TCTCGGGGATTCTTGATGTCATTTCAAAGACCACACG,

lefty1-pr-11: CGGCCAAACTGGGAAGCGAGCGGCGTTTTC,

lefty1-pr-12: TTGTTCTTTACGTTCGTTGGGATCACAAGGTTCTCCA,

lefty1-pr-13: GCGGAATTTCATTAAGTCCCAGTTTCTTCAGCAGAGC,

lefty1-pr-14: GTCTTTCATGTCTTCGTGGGTGAAACCCCTAGC,

lefty1-pr-15: CATCGCGAAAAGTGCTGCGCAAAGGAGGCA,

lefty1-pr-16: CGCGGCGCGGACTGAAGTCATCTTTTCAAG,

lefty1-pr-17: CGGCTCCTAGACCAATATTGCACCGCCTGG,

lefty1-pr-18: GTGACATCAAAGCTCTTCCAGCCAGTTTCGTGA,

lefty1-pr-19: TAGTGCGTCATGAAGATAGCTGGTCGATACAAACAGG,

lefty1-pr-20: TTTTACTAGATACATCATCGGTAGTGGCGCGCTCTC,

lefty1-pr-21: GACGACCGCGCATTTCCTCTCTCCGTAGCC,

lefty1-pr-22: GGCAGCCGCCTTTACACCTGAACGCCTGGTA,

lefty1-pr-23: ACCGGACGGCTCGATGATCCAGTACTGTGT,

lefty1-pr-24: AGTCAGAGCTCGGAAATTGATGAAGTACTGTTCCCTG,

lefty1-pr-25: AGCACATTTCACGGTCTTTGTTGTTTTCACAGTCTCC.

A 69-bp sequence with three identical imager docking sites of 20 bp interspaced by three adenosine bases was appended to the 3′ of each probe. The imager docking site for imager *pr25*^109^ was chosen, with the following sequence: ACCAATAATAACCAATAATA. The 25 probes were ordered as oligos (Sigma-Aldrich, HPLC purification), resuspended in IDTE solution (pH 8; IDT, 11050109), pooled and diluted to 4 μM concentration. Imager *pr25* (TATTATTGGTTATTATTGGT), complementary to the three docking sites on the probes, was ordered from IDT with a 5′ Atto-647 fluorophore and resuspended in IDTE buffer at 100 μM.

Embryos were fixed at 50% epiboly stage and shield stage in 4% PFA solution in PBS overnight at 4 °C. On the next day, embryos were washed 3 × 5 min in PBS and manually dechorionated. PBS was exchanged with 50%/50% PBS/methanol and then 100% methanol and stored at −20 °C. All further processing has been performed in DNA Lo-Bind 2-ml microcentrifuge tubes (Eppendorf, 0030108078). Methanol was exchanged with 50%/50% methanol/PBSTw and then washed 3 × 5 min in PBSTw. Samples were then placed at 37 °C for 30 min in 2XSSC + 0.5% TritonX-100 for permeabilization. Probe-hybridization solution was prepared fresh as follows: 2× SSC, TritonX-100 0.1%, formamide 50% (Sigma, F9037), dextran sulfate 10% (Millipore, S4030), *lefty1* probes 80 nM, RNV 10 mM (NEB, S1402S), tRNA 500 μg ml^−1^, BSA 0.1% (Invitrogen, AM2616) and ddH_2_O. A total of 200 μl prewarmed probe-hybridization solution at 37 °C was added to each sample. The sample was first placed at 60 °C for 30 min for denaturation and then incubated at 37 °C overnight. The samples were washed 4 × 30 min with 2× SSCTw (2× SSC with 0.1% Tween 20) at 60 °C and then 2 × 5 min at room temperature. Media were exchanged with PBSTw and washed 2 × 5 min and then transferred to 37 °C. Fluo-hybridization solution was prepared fresh as follows: PBS 1×, TritonX-100 0.2%, dextran sulfate 10%, imager *pr25* fluorescent probe 0.2 μM and ddH2O. Once the sample was warm, 200 μl prewarmed fluo-hybridization solution was added and the sample was subsequently incubated at 37 °C for 3 h. The sample was then washed 3 × 5 min with prewarmed PBSTw and then returned to room temperature. Samples were then permeabilized (PBS + 0.2% TritonX-100 + BSA 0.2%) for 30 min at room temperature. Primary antibody incubation with anti-E-cadherin antibody (BD, 610182, Lot 4283007) was performed overnight at 4 °C (1:200 in PBS + 0.1% Triton, 0.1% BSA). Samples were washed 6 × 10 min in PBSTw at room temperature. Secondary antibody incubation (antimouse-Alexa488, Invitrogen, A11001, Lot 2220848) was performed for 2 h at room temperature (1:500 in PBS + 0.1% Triton, 0.1% BSA) and then washed 4 × 10 min in PBSTw. Samples were stained with DAPI for 20 min (300 nM in PBSTw), then washed 2 × 5 min in PBSTw and then transferred to PBS for imaging.

### Image acquisition

All confocal imaging was performed with an upright confocal microscope Zeiss LSM 980 equipped with Airyscan 2 with Axio Examiner, with a 20× immersion objective W Plan-Apochromat 20×/1.0 Corr DIC M27 75 mm and an LD LCI Plan-Apochromat 40×/1,2 Imm Korr DIC M27 Zeiss objective. For live imaging, timelapse *z*-stack images were acquired at 10–15-min intervals with a *z* step of 2−3 μm. Images were acquired in ZEN3.3 (blue edition) software (Carl Zeiss).

For live imaging, dechorionated embryos were embedded in 0.6% low-melting point agarose (Invitrogen, cat. no. 16,520-050) in Danieau’s on a customized agarose mould (2.5% Agarose in Danieau’s) in a 60-mm dish (Greiner, 628102) and covered with Danieau’s. Mounted embryos were kept in an incubation chamber at 28.5 °C throughout acquisition and imaged with the 20× immersion objective. P-Smad2/3 immunostained samples were manually de-yolked with forceps and a tungsten needle tip, flat-mounted on glass slides and covered with a glass coverslip and then imaged with the 40× objective. β-catenin immunostained samples and smFISH samples were mounted on a customized mould of 2.5% agarose in PBS in a 60-mm dish and immobilized with a thin layer of 0.6% low-melting point agarose in PBS, covered in PBS and imaged with the 20× immersion objective. In situ hybridization samples were positioned on a customized agarose mould (2.5% agarose in PBS) in a 60-mm dish and imaged with a Leica stereoscope (Leica, M205FCA) equipped with a PLANAPO 1.0× objective set at 7× zoom and a camera (Leica DFC700T) set on colour mode and with SLI illumination, controlled with the LAS-X software (v 3.7.4, Leica Microsystems).

### Optogenetics

Embryos were kept in the dark until the selected developmental stage. To prevent photoactivation, all sample handling and mounting was performed under red-light filters (Lee Filter 106, Primary Red) to block brightfield illumination. For α-catenin–citrine degradation, *Gt(ctnna-citrine)*^*ct3a*^ homozygous embryos were microinjected with Opto-zGrad as described above. Opto-zGrad photoactivation and imaging of α-catenin–citrine degradation were carried out with 488-nm light pulses (laser power set between 3% and 6%, corresponding to an out-of-objective power of 50–104 µW) every ~2.5 min, with a *z*-stack size of ~100 µm (3.5 µm spacing) for about 30 min, and then normal imaging was performed. Dark effects were sometimes observed in a concentration-dependent manner. For Opto-RhoGEF experiments, wild-type*, MZwnt11f2/slb or MZoep* embryos were microinjected with Opto-RhoGEF–CRY2 and CIBN-CAAX mRNAs at single-cell stage and were photoactivated/acquired with a 5–6% 488-nm laser (corresponding to 86–104 µW) in a total *z* stack of 80 µm (3 µm spacing), with pulses every ~2.5 min, from sphere-onset of doming stage for about 30 min, and then normal imaging was performed.

### Data analysis and quantification

Acquired data were processed using Fiji (v2.3.0, ImageJ2)^111^, Imaris (v 10.1, Oxford instruments) and Cellpose v2 and v3^112,113^. All the data were analysed and plotted in Python (v 3.10.9) with standard packages: matplotlib (v 3.7.0), pandas (v 1.5.3 and v 2.2.2), seaborn (v 0.12.2), numpy (v 1.23.5 and v 1.26.4), imageio (v 2.34.2), scikit-image (v 0.24.0) and scipy (v 1.14.0).

#### Sebox fluorescence

Intensity profiles were generated with the plot_profile()function in Fiji with lines of thickness 110 μm in width on a maximum intensity projection (MIP) over a length of 150 μm and binned every 1 μm. The signal for each embryo was then normalized to the maximum across the *t* = 0, *t* = 30, *t* = 60, *t* = 90 and *t* = 120 min timepoints from the last YSL division.

#### α-catenin intensity

The intensity of the endogenous α-catenin fluorescence was quantified in homozygous Gt(ctnna-citrine)^ct3a^ embryos, either in wild-type (with and without Opto-zGrad) or mutant backgrounds (*MZoep* and *MZwnt11f2/slb*). For the optogenetic experiments, α-catenin–citrine intensity was measured in a 150 × 80-µm region of interest (ROI) in the margin of the blastoderm at *t* = 0, *t* = 30, *t* = 60, *t* = 90 and *t* = 120 min and normalized to the *t* = 0 min intensity of each embryo. To compare α-catenin levels between the different genetic backgrounds, fluorescence intensity was measured in a 130 × 65-µm ROI in the margin of the blastoderm at t 120 min and each condition was normalized to the mean of the controls of the same experiment.

#### Image segmentation

Blastoderm images were segmented using Cellpose v2 and v3^112^, with manual corrections being performed where necessary after inspecting the original image.

#### Reconstruction of connectivity maps and rigidity analysis

Connectivity networks were reconstructed in Fiji on ROIs of 150 × 150 µm cropped from timelapse movies, aligning the margin with the YSL at the bottom of the ROI, every 30 min from the last YSL division. As previously described in refs. ^14,16^, networks were reconstructed on the second to third layer of deep cells in the tissue. The interstitial fluid channel was binarized in Fiji via thresholding. To obtain more accurate segmentation results, the membrane channel was processed as follows: the ‘AND’ operator in the Image Calculator function was used between the membrane channel and the inverted binarized interstitial fluid channel (pixel value 0 for interstitial fluid), to convert the membrane channel areas occupied by interstitial fluid to pixel values of zero. A custom Fiji plugin based on the region adjacency graph function from the MorphoLibJ plugin^114^ was used to reconstruct connectivity networks from the cell segmentations and overlay them to the tissue and segmentation, where label adjacency was evaluated via the presence or absence of neighbouring pixels. Coordinates of the nodes are exported, as well as the adjacency matrix. The reconstruction of the networks and rigidity analysis was performed with a python version of the pebble game algorithm (pebble.py) and plotted using matplotlib^14,16^. Average connectivity was calculated as the total number of contacts (defined as described in connectivity map reconstruction) divided by the total number of cells in the image. Normalized connectivity (<*k*>) was calculated in each confocal section as connectivity divided by the maximum potential connectivity (*k*_max_) (computed as described in ref. ^14^). For the rigidity analysis along the A–V axis, the following properties of each node were extracted: *xy* coordinates, number of contacts and if the node is in the GRC, and they were then plotted in 25-µm bins. Average connectivity in this case was calculated as the total number of contacts divided by the number of cells within each bin. Owing to cropping the image to perform the analysis, connectivity at the edges of the network is underestimated because some of the neighbours of the cells at the boundaries are out of the field of view. This is relevant for connectivity of the cells at the edges of the network (0–25 µm and 125–150 µm). Please note that the critical point in connectivity for a rigidity transition is approximately four contacts per cell for average connectivity, and approximately two or three of maximum potential connectivity (*k*_c_ ≈ 0.667) for normalized connectivity^14^.

#### Relative surface tension *α*

For mapping the spatiotemporal patterns of the contact surface tensions, cell–cell contact angles were measured every 30 min from the last YSL division. Only one angle was measured for each pair of cells, and this was done in the middle of the cell volumes after inspecting the different *z* planes. A custom Fiji script was used to extract the *xy* coordinates relative to the YSL, which was marked with the line tool. A custom python code was used to calculate the *α* parameter from the angles in radians with the formula *α* = cos(θ/2), bin the data along the A–V axis every 30 µm and plot the measurements across space and time. Heat maps were generated with the seaborn library on the basis of matplotlib after transposing the data into pivot tables.

#### Interstitial fluid fraction

The interstitial fluid fraction was calculated in an ROI of 120 × 80 µm in the most marginal area of the embryos. The interstitial fluid channel was binarized via thresholding and the area fraction was then measured.

#### Tricellular contacts (closed triangles)

A custom-made Python pipeline for morpho-feature quantification in 2D ROIs of 120 × 70 µm was used^16^. In brief, segmentation masks were generated with Cellpose and manually curated after visual inspection of the membrane and interstitial fluid pockets. Using scipy.ndimage.generic_filter, a junction map was generated and the locations where three cells meet were identified. These were then normalized to the number of triangles obtained from the connectivity maps.

#### Skeletonization

Skeletonizations of 3D *z* stacks of 25 µm depth of a 100 ×50 µm ROI were performed in Fiji on the interstitial fluid channel after binarization with the Skeletonize 2D/3D()function. A maximum projection of the skeleton was done and colour-coded by depth.

#### Nodal signalling activity

To dynamically measure Nodal activity during development, Smad2–GFP mRNA-injected embryos were imaged via confocal microscopy from sphere stage for at least 3 h every 10–15 min. A region of 180 µm width was cropped, Smad2-positive nuclei were marked with the spot function in Imaris (Imaris 10.1, Oxford Instruments) at every timepoint, and the *xy* coordinates were extracted. The data were analysed in python with custom scripts: the nuclei *xy* coordinates were normalized to the lowest nucleus along the *y* axis. Data points were then binned every 20 min and aligned at the last YSL division as *t* = 0 min. Length scale was measured as the maximum distance from the YSL for each timepoint and the count as the number of positive nuclei for each timepoint. The N/C ratio was obtained by measuring in Fiji the mean grey value for the Smad2–GFP channel in the nucleus and in the cytoplasm, respectively, for each timepoint for a region of 120 µm in width, along the A–V axis. The data were analysed in python with custom scripts: the nuclei *xy* coordinates were normalized to the nucleus closest to the YSL along the *y* axis, and the ratio between nuclear and cytoplasmic signal was performed. The datapoints were then binned every 15 µm and every 30 min and aligned at the last YSL division as *t* = 0 min. The *C*_max_ and *λ* values were obtained by fitting the datapoints with the curve_fit() function within the scipy.optimize package as $y={C}_{\max }{\rm{\cdot }}{e}^{\left(-\frac{x}{\lambda }\right)}+b$, and only fits with *R*^2^ > 0.75 were taken into account.

#### Ligand gradient

The spots detection function in Imaris was used to detect the squint–GFP bright spots from 3D *z* stacks at a timepoint ~2 h after injection at the sphere stage. The *xy* coordinates of the spots were then extracted and the data were analysed and plotted with custom Python scripts. To obtain the ligand distribution, the data were binned along the *y* axis every 15 µm, expressed as distance from the YSL and plotted as density functions, after dividing the amount of spots in every bin to the total per embryo, to correct for injection differences. The *C*_max_ and *λ* values for each embryo were obtained by fitting the distribution with the curve_fit() function within the scipy.optimize package as an exponential decay, with the following function: $y={C}_{\max }\bullet {e}^{(-\frac{{x}}{\lambda })}$. The relative differences in *C*_max_ and *λ* values between the experimental conditions were preserved even when quantifying without the normalization step.

#### smFISH

For each embryo, at least ten cells in the marginal area were analysed. Both the cytoplasmatic and nuclear *lefty1* mRNA spots were counted in the middle plane of the cell in Fiji.

#### Planar projections

For visualization purposes in Fig. 1c, the blastoderm confocal *z* stacks have been planarly projected to overcome the curvature of the embryo. All the measurements and analysis have been performed on the original confocal files. For this, the Elliptical3DTransform among the bigdataviewer-biop-tools from the BigDataViewer Fiji plugin https://github.com/bigdataviewer/bigdataviewer_fiji/ has been used. In brief, the imaged embryo volume was fitted to an ellipsoid and the radial coordinates were used to unwarp the planar coordinates.

### Theoretical models

All relevant information regarding the theoretical approaches are provided in the Supplementary Theory Note. The codes used for the Nodal/Lefty/Porosity model can be found at 10.5281/zenodo.18978110 (ref. ^115^) and for the adhesion/connectivity/rigidity gradients at 10.5281/zenodo.18981645 (ref. ^116^). For generation of networks with and without connectivity gradient Lattice_Non_Uniform_Prob.py, pebble.py and lattice.py were used. The scripts Generate_random_tiling_Mono_exact.py and Run_tiling_Rand_2D_exact.py were used to create cells tilings. For creating .fe files for Surface Evolver without and with a gradient in *α*, Sim_RandomWeilbull_Sequential.py and Sim_RandomWeibull_alphagradient.py were used, respectively.

### Single-cell RNA sequencing

Acetic-Methanol (ACME), a dissociation protocol for single-cell transcriptomics, which simultaneously fixes the tissue, was used to avoid potential effects of the dissociated tissue architecture to gene expression^117^. In brief, shield-stage wild-type and *MZwnt11f2/slb* embryos were dechorionated and treated with the ACME solution. Dissociated cells were cryopreserved by adding 10% dimethyl sulfoxide and stored at −80 °C. To enrich for singlets and eliminate cell debris and aggregates, ACME-dissociated cells were stained with DAPI and cell sorting was performed by the FACS Facility at EMBL. Sample quality was assessed by evaluating cell morphology using a ZEISS LSM 980 with Airyscan 2 microscope, and RNA integrity was verified using a bioanalyzer. Single-cell RNA sequencing was carried out using the 10x Genomics platform. cDNA libraries targeting ~10,000 cells per sample were prepared using the Chromium Next GEM Single Cell 3ʹ Reagent Kits (v 3.1, 10x Genomics) following the manufacturer’s instructions. Sequencing was performed on a NextSeq2000 P2 platform with 50-bp paired-end reads by the Gene Core Facility at EMBL. Reads were processed using CellRanger (v 7.1.0, 10x Genomics). Output cell metadata and count matrix were read in and preprocessed in Monocle3 using a standard workflow: estimate_size_factors() *->* preprocess_cds () with 30 principal components, followed by sample-wise batch correction in PCA space align_cds (alignment_group = ‘Sample’, residual_model_formula_str = “~log10(n.umi)”), dimensionality reduction reduce_dimension (preprocess_method = ‘Aligned’, max_components = 2, reduction_method = ‘UMAP’). Cell clustering was performed in the batch-corrected PCA space cluster_cells (reduction_method = ‘Aligned’). Expression signatures for Nodal target genes were generated using aggregate_gene_expression () with log normalization of mean expression values across cells. De novo identification of gene modules was performed using find_gene_modules () on the subset of meso-endodermal cells, separately for wild-type and *MZwnt11f2/slb* mutant samples. Sequencing data have been deposited in the National Center for Biotechnology Information (NCBI) Gene Expression Omnibus (GEO) GSE299074, GSM9032491 and GSM9032492.

### Statistics and reproducibility

The statistical analyses were performed with GraphPad Prism 10.0. Statistical details of experiments are reported in the figures and figure legends. Sample sizes are provided in the figure legends; no statistical test was used to determine sample size, but our sample sizes are similar to those reported in previous publications^14,19^. The biological replicate is defined as the number of embryos. No inclusion or exclusion criteria, randomization or blind allocations were applied, and all analysed samples were included. Unless differently stated in the figure legends, the graphs show mean ± s.e.m., and the error bars are calculated and shown on the basis of the number of cells or embryos, as indicated. The statistical test used to assess significance is stated in the figure legends. For comparisons between two groups, a two-tailed Student’s *t*-test was used for parametric distributions with equal variances. For multiple pairwise comparisons, an analysis of variance followed by Dunnett’s test was used for parametric distributions, while a Kruskal–Wallis test followed by Dunn’s test with Dunnett’s adjustment (for pairwise comparisons) was applied for non-parametric distributions.

### Reporting summary

Further information on research design is available in the Nature Portfolio Reporting Summary linked to this article.

## Online content

Any methods, additional references, Nature Portfolio reporting summaries, source data, extended data, supplementary information, acknowledgements, peer review information; details of author contributions and competing interests; and statements of data and code availability are available at 10.1038/s41556-026-01954-4.

## Supplementary information

Supplementary Information Supplementary Theory Note (model and theory).

Reporting Summary

Peer Review File

Supplementary Movie 1 Mapping tissue rigidity during meso-endoderm specification. Exemplary timelapse movie of a transgenic embryo expressing eGFP under the *sebox* promoter, labelled with membrane, nuclei and interstitial fluid markers, with overlaid rigidity map in a 150–180-μm region including parts of the specification zone and the overlaying pluripotent blastoderm. The black network is the connectivity map and the red network is the Giant Rigid Cluster marking tissue rigidity. Scale bar, 50 μm.

Supplementary Movie 2 Nodal signalling dynamics in different experimental conditions. Exemplary timelapse movies from the margin of wild-type, *MZoep*, *MZlefty1/2*, *MZwnt11f2/slb* and *MZwnt11f2/slb* +OptoRhoGEF embryos labelled for Smad2–GFP and dextran-647 for interstitial fluid. Magenta arrowheads indicate Smad2-positive nuclei, white open arrowheads indicate open interstitial gaps (high porosity) and filled white arrowheads indicate close interstitial gaps (low porosity). The dashed line indicated the length scale of Nodal signalling. Scale bar, 25 μm.

## Source data

Source Data All Figures Statistical source data for all main and Extended Data figures.

## Data Availability

The single-cell sequencing datasets generated in this study are publicly available with the following Gene Expression Omnibus (GEO) accession codes: GSE299074, GSM9032491 and GSM9032492. The *lefty1* coding sequence used to design the smFISH probes is available under accession code ENSDART00000019196.7. Any additional data can be provided by the corresponding author of this study. Source data are provided with this paper.

## Code availability

Code used to obtain numerical solutions for Nodal–Lefty–rigidity dynamics is available via Zenodo at 10.5281/zenodo.18978110 (ref. ^115^). Code used to generate network–rigidity dynamics is available via Zenodo at 10.5281/zenodo.18981645 (ref. ^116^).
