# Supplementary material for: Tissue rigidity phase transition shapes morphogen gradients
Source: Nat Cell Biol. 2026 May 14;28(6):1191–203. doi: 10.1038/s41556-026-01954-4 (PMC13278970; doi:10.1038/s41556-026-01954-4)
Supplement: Supplementary file 1 — Supplementary Theory Note (model and theory). [file 41556_2026_1954_MOESM1_ESM.pdf]

nature cell biology

Supplementary information https://doi.org/10.1038/s41556-026-01954-4

Tissue rigidity phase transition shapes morphogen gradients

In the format provided by the authors and unedited

Table of contents:

Supplementary Note……………………………………………………………………………………………………2

1. Theoretical framework…………………………………………………………………………………………2

1.1. Coarse-grained concentrations in a porous environment…………………………………2

1.2. Coupled dynamics of Lefty and Nodal……………………………………………………………2

1.3. Scaling of degradation rates and diffusion constants with tissue porosity…………3

1.4. Regulation of cell-cell adhesion and the relative surface tension α via Nodal……..5

1.5. Derivation of porosity as a function of α……………………………………………………5

1.5.1. Triangular gaps……………………………………………………………………………6

1.5.2. Quadrilateral gaps………………………………………………………………………7

1.5.3. Approximation for the porosity index……………………………………………8

2. Numerical Solutions………………………………………………………………………………………………8

2.1. Quantifications……………………………………………………………………………………………9

2.2. Parameter choices………………………………………………………………………………………11

2.3. The role of Nodal relay…………………………………………………………………………………12

3. Impact of diffusivity on the morphogen amplitude and adhesion…………………………………15

4. Simulations of cell networks and cell tiling with varying connectivity and adhesion………..25

4.1. Construction of networks with connectivity gradient……………………………………….16

4.2. Construction of in-silico tilings with adhesion gradient……………………………………16

4.3. Rigid cluster analysis……………………………………………………………………………………………17

1 Theoretical framework

In this section we lay out the theoretical framework we have developed to couple Nodal-Lefty reaction diffusion dynamics with tissue porosity. We further present derivations that couple the relative surface tension between cells to tissue porosity.

1.1 Coarse-grained concentrations in a porous environment

We base our analysis on a model for Lefty and Nodal dynamics in the zebrafish blastula that has been proposed as an inhibitor-activator system and previously fitted to experimental data in [1]. We couple this model with a spatially varying tissue porosity as described below. We assume that all quantities are averages over the thickness of the tissue layer (3-5 cells thick) and ignore any dependence on the curvature of the embryo, as this remains constant during the time window and the region of interest.

We introduce coarse-grained concentrations c, locally averaged over the extracellular and cellular regions (see Fig SN1). The time evolution of a coarse-grained concentration field $c(\mathbf{x}, t)$, with $\mathbf{x} = (x, y)$ the coordinates within the region of interest, is generally given by an advection-diffusion equation [2]

$$\partial_t c = \nabla \cdot (D\nabla c - \mathbf{v}c) + \mathcal{R}, \tag{1}$$

with a diffusive flux $-D\nabla c$, an advection velocity $\mathbf{v}$, and reaction terms denoted by $\mathcal{R}$. Although we do not consider hydrodynamic flows, the spatially heterogeneous porosity $\phi(\mathbf{x})$ requires a non-zero apparent advection velocity as derived below.

The coarse-grained concentration is defined through the porosity as

$$c = \phi c_{ext} + (1 - \phi)c_{int}. \tag{2}$$

If the extracellular and intracellular concentrations, given by c_{ext} and c_{int} respectively, are related by linear reactions (such as endo- and exocytosis with constant rates) and those equilibrate on time scales faster than the tissue-wide morphogen dynamics, we have $c_{int} = \beta c_{ext}$ and

$$c = (\beta + (1 - \beta)\phi)c_{ext}. \tag{3}$$

In the absence of reactions at the coarse-grained level, $\mathcal{R} = 0$, at steady state extracellular (and intracellular) concentration must be uniform in space $c_{ext} = c^0_{ext} = const.$ and fluxes must vanish,

$$D\nabla c_0 = \mathbf{v}c_0. \tag{4}$$

With $c_0(\mathbf{x}) = (\beta + (1-\beta)\phi(\mathbf{x}))c^0_{ext}$, this determines the form of an apparent velocity, resulting from gradients in the porosity $\phi(\mathbf{x})$,

$$\mathbf{v} = D\nabla \log\left(\beta + (1 - \beta)\phi\right). \tag{5}$$

This contribution to the transport equation constitutes an apparent flux towards regions of higher porosity. For $\beta = 0$, we have $\mathbf{v} = D\nabla \log(\phi)$, which is the result for a porous medium with impermeable obstacles [3]. Conversely, for $\beta = 1$, i.e. $c_{int} = c_{ext}$, the velocity term vanishes.

1.2 Coupled dynamics of Lefty and Nodal

We reduce our description of Nodal-Lefty dynamics in the blastoderm to a continuous 1D domain along the direction of the morphogen gradients, $x \in [0, l]$, with $x = 0$ denoting the border with the YSL and l the length of the measurement window. This reduction is justified because we observe no gradients of the morphogens in the circumferential (y) direction. Starting from equations (1) and (5) for the concentrations of Nodal $N(x)$ and Lefty $L(x)$ we now add the reaction terms following the inhibitor-activator interactions [1]. We then arrive at a coupled system of partial differential equations ,

$$\partial_t N = \partial_x(D_N\partial_x N - D_N N\partial_x \log(\beta + (1 - \beta)\phi)) - k_N N - k_{NL} N\frac{1}{1 + (L_N/L)^2} + \nu(N) + s_0\delta(x), \tag{6}$$

$$\partial_t L = \partial_x(D_L\partial_x L - D_L L\partial_x \log(\beta + (1 - \beta)\phi)) - k_L L + \nu_L(N), \tag{7}$$

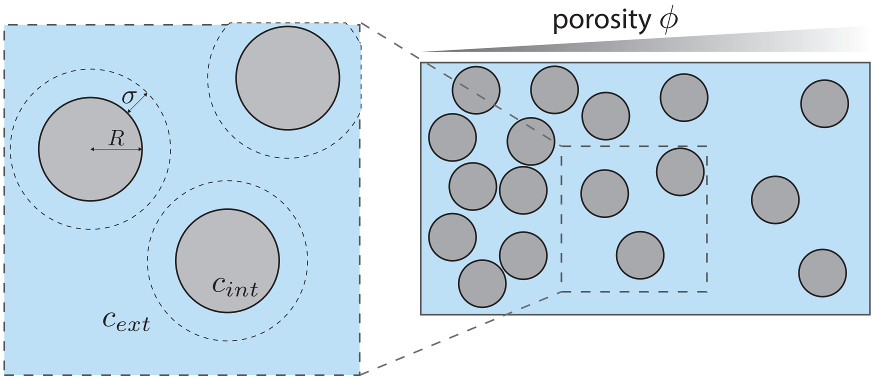

Figure SN1: We model the tissue as a porous material with spatially graded porosity $\phi(\mathbf{x})$. Cells are denoted by grey circles and assumed to have a constant radius R. The porosity is the locally-averaged fraction of extracellular space, e.g. in a small region of the tissue having area A_c containing N_c cells it is given by Equation (11). We further denote by c_{ext} and c_{int} the extracellular and intracellular concentrations of morphogen, which are locally averaged to obtain c (see Eq. (2)). The morphogen uptake into a cell happens from a ring of size σ around the cell.

where both morphogens diffuse with diffusivities $D_{N,L}(\phi)$ and are degraded linearly with rates $k_{N,L}(\phi)$ [4]. We also include Lefty-mediated inhibition of Nodal (k_{NL}), self-activation of Nodal via relay [5] ($\nu(N)$) and Nodal-mediated Lefty activation ($\nu_L(N)$) [6, 7]. There is an actively regulated influx of Nodal molecules from the YSL into the tissue which we model by a delta-peak of Nodal production at $x = 0$ with a magnitude s_0. At the tissue boundaries the remaining diffusive fluxes are vanishing,

$$\partial_x L|_{x=0} = 0, \quad \partial_x N|_{x=l} = \partial_x L|_{x=l} = 0. \tag{8}$$

For the activation terms we assume a Hill-type function

$$\nu(N) = \sigma_N \frac{1}{1 + (N_a/N)^{m_N}}, \tag{9}$$

$$\nu_L(N) = \sigma_L \frac{1}{1 + (N_L/N)^{m_L}}, \tag{10}$$

with activation strengths $\sigma_{N,L}$, Hill coefficients $m_{N,L}$ and activation thresholds $N_{a,L}$. We assume that the coarse-grained concentrations used here capture signalling levels, e.g. as measured by Smad2-positive nuclei (see main text Fig. 2 d,e).

1.3 Scaling of degradation rates and diffusion constants with tissue porosity

To connect the transport and kinetic parameters to the area-averaged interstitial fluid fractions measured experimentally (main text Fig. 3 d), we consider a 2D projection of the tissue.

The porosity $\phi = \phi(\mathbf{x})$ is defined as

$$\phi = \frac{A_{tot} - N_c A_c}{A_{tot}}, \tag{11}$$

that is the fraction of extracellular space in a small region of the tissue of area A_{tot} centred at $\mathbf{x}$ and containing N_c cells of area A_c. Note that this 2D porosity is equivalent to the interstitial fluid fraction (IFF) measured in the experiments throughout the article. From the literature on porous materials we expect that porosity affects the coarse-grained transport properties of morphogens in a tissue [8, 9, 10].

For many types of porous materials it has been shown that effective diffusivity follows an approximately linear scaling with porosity [8, 11, 3, 12]

$$D \propto \phi. \tag{12}$$

Deviations from this scaling are possible at small porosities, depending on the specific geometry at the scale of the obstacles (i.e. cells) [13]. In the absence of an exact scaling relationship for the cell geometry that we observe in experiments, we assume that the effective diffusivity for Nodal and Lefty follows a linear relationship (see also main text Supplementary Fig. 3p)

$$D_{N,L}(\phi) = \frac{D^0_{N,L}}{\phi_0}\phi, \tag{13}$$

where $D^0_{N,L}$ is the value of effective diffusivity at $\phi = \phi_0$ (the initial value of porosity).

We now discuss how effective degradation and production terms scale with porosity. Consider N_c cells of radius R (see Figure SN1). The number of ligands internalised into the cells per unit of time is $k_{int} N_c \sigma R c_{ext}$, where c_{ext} is the extracellular concentration of ligands and internalisation happens with a rate k_{int} from a ring of width σ around the cell membrane. Using Eq. (11) this can be rewritten as

$$k_{int}\sigma N_c R c_{ext} = k_{int}\sigma \frac{A_{tot}}{A_c}(1-\phi) R c_{ext} \tag{14}$$

$$= k_{int}\sigma \frac{A_{tot}}{\pi R}(1-\phi) c_{ext}, \tag{15}$$

where in the last step we used $A_c = \pi R^2$. We are seeking an expression in terms of the coarse-grained concentration c. At small porosities and $\beta << 1$ we can write Eq. (3) as $c \approx (\phi + \beta) c_{ext}$. Then the total loss of ligands per unit of time can be written as

$$k_{int}\sigma \frac{A_{tot}}{\pi R} \frac{1-\phi}{\beta+\phi} c \tag{16}$$

and one identifies the effective degradation as the prefactor of c, with the scaling

$$k_{eff} \propto \frac{1-\phi}{\beta+\phi}. \tag{17}$$

The effective degradation is thus an increasing function of the cell packing density. In the linear degradation terms in Eq. (6) and (7) we set

$$k_N = k^0_N \frac{\beta+\phi_0}{1-\phi_0} \frac{1-\phi}{\beta+\phi}, \tag{18}$$

$$k_L = k^0_L \frac{\beta+\phi_0}{1-\phi_0} \frac{1-\phi}{\beta+\phi}, \tag{19}$$

with $k^0_{N,L}$ the values at $t = 0$. In the limit $\beta \ll \phi$ and $\phi \ll 1$, we obtain $k_{eff} \propto 1/\phi$ and so effective degradation would increase inversely proportional to the cell distance [14]. Note, that for any $\beta \neq 0$ the expressions (18) and (19) remain finite for $\phi \to 0$.

We can obtain an expression for β, considering also degradation of internalised molecules with rate k_o,

$$\beta = \frac{k_{int}}{k_o} \frac{2\sigma}{R}. \tag{20}$$

A similar scaling can be obtained for effective production. If one cell produces ν_c molecules per time, then in the tissue we will have $N_c \nu_c$ molecules produced per time. Therefore the effective production per area is

$$\nu_{eff} = \frac{N_c \nu_c}{A_{tissue}} \tag{21}$$

Since $A_{tissue} = N_c A_c + A_{ext}$, we can express $N_c = (A_{tissue} - A_{ext})/A_c$ and

$$\nu_{eff} = \frac{\nu_c}{A_c}\left(1 - \frac{A_{ext}}{A_{tissue}}\right) = \frac{\nu_c}{A_c}(1-\phi). \tag{22}$$

If the cell area A_c stays constant as ϕ changes, then the effective production scales as $(1-\phi)$ with porosity. Thus, the higher the packing, the more molecules per area of tissue are produced. In equations (9) and (10) we set

$$\sigma_N = \frac{\sigma_N^0}{1-\phi_0}(1-\phi), \tag{23}$$

$$\sigma_L = \frac{\sigma_L^0}{1-\phi_0}(1-\phi), \tag{24}$$

where $\sigma_{N,L}^0$ are the values set at $t = 0$.

1.4 Regulation of cell-cell adhesion and the relative surface tension α via Nodal

We now introduce the dynamics of the local porosity as a function of the Nodal concentration. As shown in the main text (Fig. 2a-c and Supplementary Fig. 3d, e) cell-cell adhesion strength is increasing with higher local Nodal levels. We introduce an effective, Nodal-mediated adhesion variable E, whose dynamics follows

$$\partial_t E = \sigma_E \frac{1}{1 + N_E/N} - k_E E, \tag{25}$$

with an activation threshold N_E and degradation rate k_E that sets the time scale of adhesion build up. We set $E(x, t = 0) = 0$, such that this adhesion parameter captures only the additional adhesion strength beyond the baseline level at $t = 0$. Note that this is an effective parameter that captures local adhesion factors as a function of Nodal. For $N \gg N_E$, we would obtain the steady state value $E^* = \sigma_E/k_E$, which in turn is determined by the range of the relative surface tension α (see below).

We then describe the impact of this effective adhesion strength on α (introduced in the main text) as an algebraic relationship, such that α decreases with E as

$$\alpha = \frac{1}{E + \alpha_0^{-1}}, \tag{26}$$

such that the simulation starts with the initial value of $\alpha(x, t = 0) = \alpha_0 = 0.881$ for all positions x as measured in experiments (see main text, Fig 1g'). We set the lower bound to be $\alpha_{min} = 0.7$, so that α varies in the experimentally relevant range (see main text, Fig. 1f'). This would be reached at maximal adhesion, and therefore sets $E^* = \alpha_{min}^{-1} - \alpha_0^{-1} \approx 0.3$ and $\sigma_E = E^* k_E = 0.3 k_E$. In this way Eq. 26 is constrained by experimental measurements and does not introduce new free parameters in our model. Note that parameters that define E dynamics (i.e. k_E and N_E) can then be tuned to modulate the α dynamics as a function of Nodal. Finally, α is updated in time irreversibly, i.e. at each position it can only decrease in time. This reflects the stability of adhesive contacts between cells.

1.5 Derivation of porosity as a function of α

To link tissue porosity to the relative surface tension α, we consider that the porosity of a tissue can be split into the individual contributions of the different gaps between cells, depending on the cells defining it. We have triangular gaps (surrounded by 3 cells), quadrilateral gaps (surrounded by 4 cells), pentagonal gaps (surrounded by 5 cells) and so on. Therefore, the porosity index $\phi(\alpha)$, can be rewritten as:

$$\phi(\alpha) = \phi_0 + \phi_\triangle(\alpha) + \phi_\square(\alpha) + \ldots \quad , \tag{27}$$

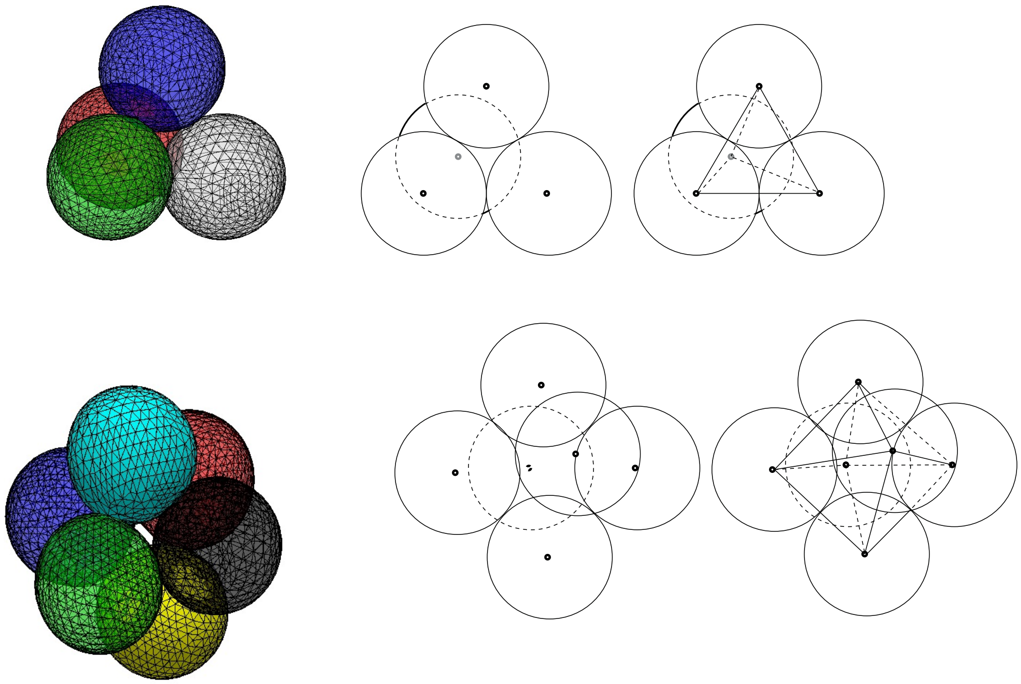

Figure SN2: Packings in dense tissues –approximated by FCC crystallographic structures– show triangular, tetrahedral, octahedral and quadrilateral gaps. In 2D projections we consider only triangular and quadrilateral gaps.

where ϕ_0 is the minimum porosity, imposed by physiological constraints, and $\phi_{\triangle}(\alpha)$, $\phi_{\square}(\alpha)$ the contribution of each gap type as a function of α. To characterize the different terms, we will consider circles of radius $R = 1$. The center of masses of adjacent circles in the geometric arrangement defining the gap will be separated by distance $\ell = 2R$. This distance will be kept constant throughout all the forthcoming developments. The area occupied by the cells will be computed by rescaling the radius of the circles –thereby formally triggering overlap– by an abstract scaling factor $\xi \geq 1$. The overlap will define an angle that can be related to the scaling parameter ξ and the relative surface tension parameter α derived in [15]:

$$\alpha = \frac{1}{\xi} \ . \tag{28}$$

We assume that the circles for $\alpha = 1$ have radius $R = 1$ and $\xi = 1$. In the following section we will study the porosity from the analysis of the area covered by triangular and quadrilateral gaps. We consider these gaps because we approximate the structure of the 3D tissue to be close to the *face-centered cubic* (FCC) packing [16]. The projection of the FCC packing structure in 2D leaves triangular and quadrangular gaps –see Fig. (SN2) and Fig. (SN3).

1.5.1 Triangular gaps

For each triangular gap, we consider the surface of triangle formed by the three centers of mass of three cells and how this is getting filled along the increase of ξ. Considering the fact that the triangle contains 1/6 of each of the 3 cells and the 1/2 of the intersection surface shared by each pair of cells, we have that the

extracellular relative surface, ϕ, occupied by the cells within the triangle is:

$$\phi_{\triangle} = 1 - \frac{1}{\mathrm{A}(\triangle)}\left(\frac{1}{2}\int_S dS - \frac{3}{2}\int_{\cap} dS\right) \ ,$$

This leads to an equilateral triangle of side 2, being its overall surface of the $\mathrm{A}(T) = \sqrt{3}$. After applying a rescaling operation $R \to \xi R$, the other terms read:

$$\int_S dS = \pi\xi^2 \ ; \qquad \int_{\cap} dS = 2\left(\xi^2 \arccos\left(\frac{1}{\xi}\right) - \sqrt{\xi^2 - 1}\right) \ .$$

In terms of α, using that $\alpha = \frac{1}{\xi}$:

$$\phi_{\triangle}(\alpha) = 1 - \frac{\sqrt{3}}{\alpha^2}\left(\frac{\pi}{6} - \arccos(\alpha) + \alpha\sqrt{1-\alpha^2}\right) \ , \quad (\alpha \geq \alpha_c) \ . \tag{29}$$

Triangular gaps are closed at $\alpha_{c_1} \approx 0.866$. Even closed, there might remain some space open allowing the transport of morphogen molecules. This is grasped by the constant ϕ_0. As we can observe in figure (SN3), even allowing little openings, the closing of triangular gaps at α_{c_1} implies a collapse or drastic reduction of the connectivity of the interstitial network.

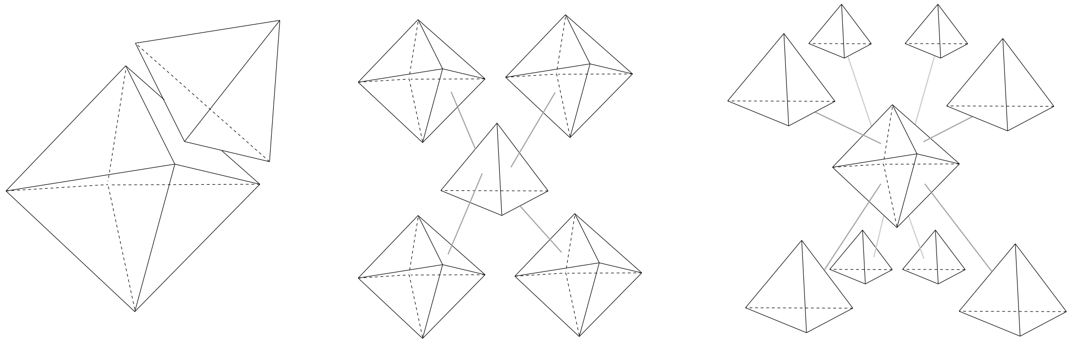

Figure SN3: Local structure of the *gap network* of a Face-Centered Cubic (FCC) packing. Vertices describe the center of mass of the cells, connections describe cell-cell contacts. Interstitial channels cross the triangular faces. When they are closed at $\alpha_{c_1} \approx 0.866$ the whole network either collapses or, suffers a drastic reduction of its connectivity. However, octahedral structures, with a square-like configuration in the center may retain certain fluid that may disappear below $\alpha_{c_2} \approx 0.707$, where, in force balance, no gaps are allowed any more in densely, FCC-like packings.

1.5.2 Quadrilateral gaps

Using analogous reasoning, we can derive the relation accounting for the contribution of quadrilateral gaps. The general form will be:

$$\phi_{\square} = 1 - \frac{1}{\mathrm{A}(\square)}\left(\int_S dS - 2\int_{\cap} dS\right) \ .$$

The area of the square will be $A(\square) = 4$, leading to:

$$\phi_{\square}(\alpha) = 1 - \frac{1}{\alpha^2}\left[\frac{\pi}{4} - \left(\arccos(\alpha) + \alpha\sqrt{1-\alpha^2}\right)\right] \ .$$

Using the relation described in equation (28), we can infer the α value at which quadrilateral gaps are closed, by just computing the re-scaling parameter ξ needed to reach the centroid of the square. A straightforward

calculation leads to $\xi = \sqrt{2}$. Therefore, we identify a second critical point below which, in a densely packed tissue, no interstitial gaps are expected, $\alpha_{c_2} \approx 0.707$. Using the same reasoning, we observe that the central point of a tetrahedron is reached at α_{c_2}. Consistently:

$$\phi_{\triangle}(\alpha_{c_1}) = \phi_{\square}(\alpha_{c_2}) = 0 \; .$$

1.5.3 Approximation for the porosity index

In a densely packed tissue, the main contributions to porosity are driven by square-like gaps and triangular-like gaps –see figures (SN2) and (SN3). We neglect the role of tetrahedral gaps, as they reveal vanishingly small in 2D projections. In these regards, comparison of equation (27) with real data shows a simple form:

$$\phi(\alpha) \approx \phi_{min} + \phi_{\triangle}(\alpha) + \phi_{\square}(\alpha) \; . \tag{30}$$

That is just the sum of the triangular and quadrilateral contributions. We assume a contribution of $\phi_{min} = 10^{-3}$ for the minimum porosity attainable. This is consistent with experimental measurements in highly rigid and packed tissues (Fig. 3d in the main text). With this offset we take into account that diffusive transport, albeit slowed down considerably [17], never completely vanishes in a packed tissue. Expression (30) closes the system of equations and provides a feedback mechanism from Nodal signal, via $\phi(\alpha)$, to the transport and kinetic parameters in the Nodal dynamics.

2 Numerical solutions

We solve equations (6), (7), (25) together with the relations (26) and (30) numerically on $[0, l]$ with a uniform spatial grid with spacing $dx = 1$. Spatial derivatives are discretised and equations (6), (7), (25) are integrated in time using the Euler scheme with $dt = 0.9(dx)^2/(2\max\{D_N, D_L\})$, according to the stability criterion for diffusion.

We begun by basing our analysis within the parameter ranges identified in [1]. Note, that there the parameter fits were performed using Nodal and Lefty profiles at the time of 50% epiboly (t=120min). Here, we instead consider both the intermediate dynamics and the steady state profiles.

We initially keep the diffusivities, degradation and production rates for Lefty and Nodal constant in time and space. In this case, the Nodal-Lefty system (Equations (6)-(7)) displays a non-monotonic Nodal dynamics (Fig. SN4 a), reminiscent of the one we find in experimental quantifications shown in the main text. At steady state, there is a Nodal gradient with a physiological range and a basically flat Lefty profile. At intermediate times, the Nodal gradient extends further into the tissue, but retracts as the amount of Lefty increases.

We then consider the full system of equations including the feedback through porosity, with parameters given in Table 1. We find that generally this enhances the non-monotonic dynamics of Nodal, with Nodal gradients retracting to a sharp profile (i.e. with reduced decay length) with higher levels of Nodal localised near the source (Fig. SN4 a versus SN4 b).

To quantify the spatiotemporal dynamics of the Nodal range we follow the method outlined below (see Fig. SN4 b):

- Define $\lambda_{1/2}(t)$ as the distance at which the concentration reaches $N(x = 0, t)/2$ for each time t. This is a dynamical variable that captures the range of the Nodal gradients over time.
- Find the time $\tau = \text{argmax}_t \, \lambda_{1/2}(t)$. This time point indicate the time at which the Nodal gradient is the widest and so the time point just before the Nodal gradient begins to retract.
- Define $N_{max} = N(x = 0, \tau)$, that is the maximum Nodal concentration at the time point when the Nodal range is maximised. We then quantify the range of the Nodal profile over time as the position x_N at which the gradient reaches the p fraction of that value, i.e. $N(x_N) < pN_{max}$, with $p = 0.1, 0.2, 0.3$.

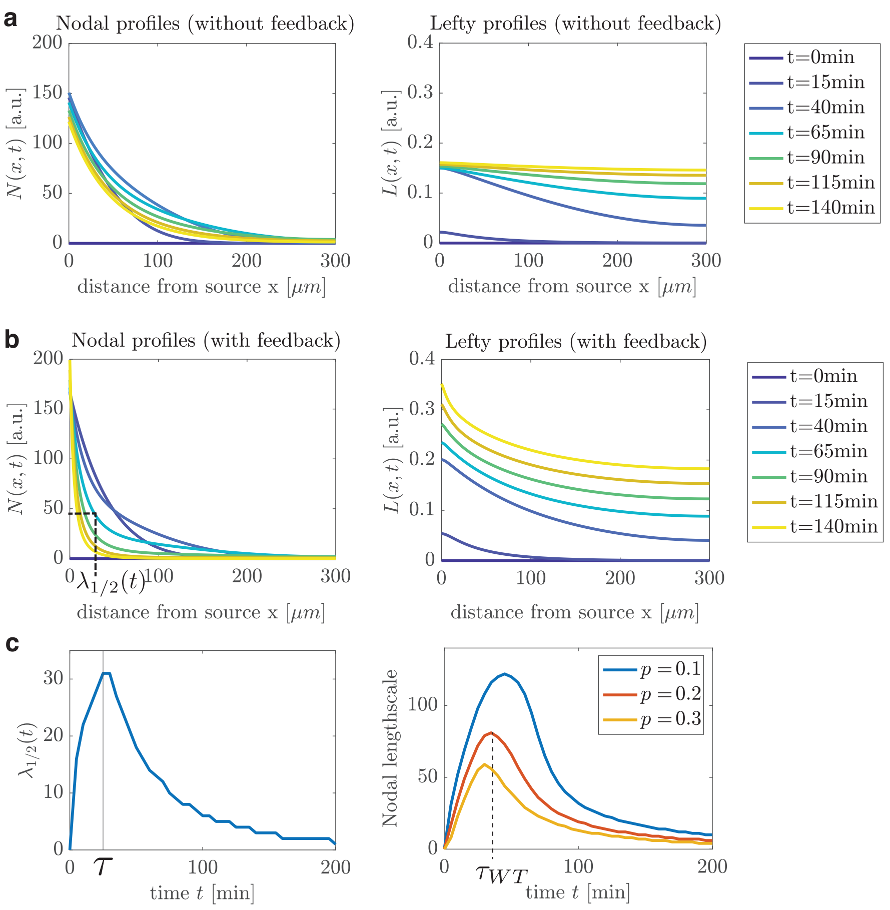

Figure SN4: **(a)** Dynamics of Nodal and Lefty with constant diffusivities and degradation and production rates (i.e. without feedback). **(b)** Dynamics of Nodal and Lefty with feedback through porosity. c) Quantification of the non-monotonic dynamics of the Nodal range, with the time of Nodal peak τ_{WT} defined on the $p = 0.2$ curve. Parameters are as given in table 1, but with $\sigma_E = 0$ (i.e. porosity dynamics is turned off).

- We normalize time by defining τ_{WT} to be the time of the peak in the Nodal length scale profile and setting $\tau_{WT} = 1$.

This approach allows us to assess the position beyond which the Nodal signalling levels drop below a pre-defined threshold. This method of quantifying the Nodal range is designed to be comparable to the experimental quantifications in the main text where we quantify the Nodal range by measuring the number and extend of Smad2 positive nuclei, presumably only detecting nuclei that have sufficiently high levels of Smad2. The quantifications presented in Fig. 3 in the main text take $p = 0.2$. Note that the value of N_{max} is quantified from the WT numerical solutions (with feedback) and the same value of N_{max} is used to define the lower threshold for signal detection in WT and wnt11 solutions (with and without feedback respectively).

2.1 Quantifications

We now compare simulations that represent the following experimental conditions: WT (full model; labelled *with feedback*), MZwnt11f2/slb mutant (E, α, ϕ dynamics turned off: $\sigma_E = 0$; labelled *without feedback*), MZlefty1/2 mutant (L production turned off: $\sigma_L = 0$; labelled *without lefty*).

Parameter	Numerical value	Explanation
D_N^0	1.95 $\mu m^2/s$	Nodal effective diffusivity at $t = 0$; Average of $0.7\mu m^2/s$ (Cyclops) and $3.2\mu m^2/s$ (Squint) from [4]
D_L^0	15.0 $\mu m^2/s$	Lefty effective diffusivity at $t = 0$; Average of $11.1\mu m^2/s$ (Lefty1) and $18.9\mu m^2/s$ (Lefty2) from [4]
l	$300\mu m$	size of experimental measurement window
k_N^0	$1.25 \times 10^{-6} s^{-1}$	Nodal eff. degradation rate at $t = 0$
k_L^0	$7.5 \times 10^{-7} s^{-1}$	Lefty eff. degradation rate at $t = 0$
k_{NL}	$10s^{-1}$	Lefty-Nodal inhibition parameter; range $10^{-5} - 10^{-1}$ in [1]
N_a	31.62 a.u.	Nodal production activation threshold
N_L	300 a.u.	Lefty production activation threshold
L_N	15.8 a.u.	Lefty-induced inhibition threshold
σ_N	10^{-2} a.u.	Nodal (relay) production strength
σ_L	1.2×10^{-2} a.u.	Lefty production strength
k_E	2×10^{-4}	sets timescale of adhesion strength build-up
N_E	23.71	$0.75 \times N_a$
σ_E	5.88×10^{-5}	$\sigma_E = E^* k_E$
E^*	0.2941	$E^* = \alpha_{min}^{-1} - \alpha_0^{-1}$
α_0	0.881	$< \alpha(x) > (t = 0)$ from experiment (WT, see main text)
α_{min}	0.7	minimal α from experiment
β	0.1	$\beta = c_{int}/c_{ext}$
m_N	2	Hill-coefficient in Nodal relay
m_L	8	Hill-coefficient in Lefty production
s_0	5	source of Nodal at $x = 0$

Table 1: Numerical values of parameters for Figure SN4.

Parameter	Numerical value
k_N^0	$1.5195 \times 10^{-6} s^{-1}$
k_L^0	$7.4653 \times 10^{-7} s^{-1}$
k_{NL}	$10.3034\, s^{-1}$
N_a	31.6228 a.u.
N_L	200 a.u.
L_N	19.1507 a.u.
σ_N	10^{-2} a.u.
σ_L	4.6539×10^{-4} a.u.
k_E	2.3082×10^{-4}

Table 2: Numerical values of parameters for main text Figures 3 and 4 (parameters not listed here are the same as in Table 1).

Throughout our analyses we compare model dynamics to the experimental observations by quantifying the following measures:

- the Nodal range as defined above, with $p = 0.2$
- time τ_{WT} of Nodal range peak in the WT simulation and normalise time t relative to this
- the average porosity $<\phi>= \frac{1}{l_1}\int_0^{l_1}\phi(x)dx$, $l_1 = 50\mu m$, corresponding to the IFF measurement close to the YSL.
- the total production of Lefty and Nodal in the tissue over time: $N^{prod}(t) = \int_0^L dx \frac{\sigma_N}{1+(N_a/N(x))^{m_N}}$, $L^{prod}(t) = \int_0^L dx \frac{\sigma_L}{1+(N_L/N)^{m_L}}$.
- spatiotemporal profiles of Nodal $N(x,t)$, which we compare to the experimental profiles of Smad2 N/C ratios

2.2 Parameter choices

Table 2 shows the parameters used for the theoretical model results shown in figures 3 and 4 in the main text. Although the expansion of the Nodal gradient and a delay in Nodal termination appear to be general features of the theoretical set-up with feedback between porosity and Nodal, we have identified some dependencies of these behaviours on parameter choices.

The observed features depend on the following relations between the activation thresholds for the three key model components that depend on the local Nodal levels: $N_E < N_a < N_L$. In other words, as the Nodal profile grows, the following temporal sequence drives the dynamics we observe

- first, the adhesion strength begins to increase, leading to a drop in the porosity near the margin
- second, the Nodal relay is activated, extending the Nodal gradient further into the tissue
- third, Lefty levels start to rise and eventually cause the Nodal profile to retract. The porosity-mediated localisation of both Nodal and Lefty near the source occur on a similar time scale, in order to enhance the inhibiting effect of Lefty.

For the Lefty production to be responsive to the porosity-induced changes of Nodal dynamics, the activation threshold for Lefty, N_L, needs to lie approximately at the maximum Nodal values achieved in the no-feedback (MZwnt11f2/slb) simulations. In this way, the porosity-mediated localisation of Nodal at the source enhances local Lefty production. This, in turn, reinforces the biochemical feedback between Lefty and Nodal (as shown by L-N degradation term).

Further, parameters are chosen to ensure that in the absence of Lefty, the Nodal gradient extends throughout the entire tissue (see Fig. SN5), i.e. the porosity change alone is not sufficient to cause Nodal retraction. Imposing this constraint in our theoretical model is motivated by our experimental quantifications of the Smad2 positive nuclei in Lefty mutants, where Smad2 positive nuclei extend to the entire tissue (Fig. 2e in the main text).

To improve our parameter estimation we used the Mesh Adaptive Direct Search algorithm (MADS), designed for difficult blackbox optimization problems [18]. These issues occur when the functions defining the objective are the result of costly computer simulations, which in this work arise since we are solving the dynamics of Eq.(6), (7), (25) numerically. The loss function was defined as the sum of two contributions. First, the mean square error between the experimentally measured Smad2 positive nuclei distance from the YSL over time in WT and MZwnt11f2/slb conditions (Fig. 3g and h in the main text) and the corresponding range of the Nodal signalling gradient with and without feedback that we computed as outlined in the previous subsection. Second, the loss function accounts for the mean square error between the α quantifications in space and time in the WT data and the corresponding values of α computed numerically; Supplementary Fig. 3f cited in the main text shows experimental data and best theoretical fit found by the optimizer for

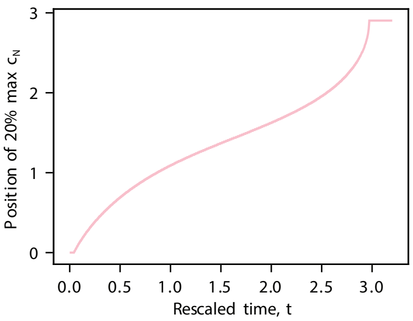

Figure SN5: The range of Nodal length scale in numerical solutions without Lefty ($\sigma_L = 0$) for the set of parameters used to produce all main text figures. The max C_N used here is based on the case with feedback in Fig. 3f (main text). Parameters: see Table 2. The length scale reaches saturation, i.e. Nodal extends through the entire tissue.

$\alpha(x, t)$. These two mean squared errors were individually normalized so that their contributions were equal. This was run locally to the parameter sets we had identified from previously published work and using the analysis above.

2.3 The role of Nodal relay

Nodal has been proposed to disperse through diffusion of ligands secreted from the YSL and a relay mechanism whereby Nodal signalling activates its own transcription and secretion [5]. We have therefore implemented both of these processes to our theoretical framework and analysis.

Previous work shows that a relay mechanism can lead to production in the entire tissue in certain parameter ranges [19]. To estimate the relay strength σ_N for which this would happen in our model and define parameter regimes where relay operates, we consider the Nodal equation with $m_a = 1$, in the absence of Lefty and the external source,

$$\partial_t N = D_N \partial_x^2 N - k_N N + \sigma_N \frac{N}{N_a + N}. \tag{31}$$

The steady state equation has two constant solutions

$$N_1 = 0, \quad N_2 = \frac{\sigma_N - k_N N_A}{k_N}. \tag{32}$$

However, $N_2 > 0$ (i.e. the non-trivial solution exists) only if $\sigma_N > k_N N_a$, which provides an estimate for the critical activation strength beyond which the entire tissue will be producing, also in the case of a non-zero source.

To investigate whether our findings hold true, independently of the relative contribution of diffusive versus relay-driven mechanisms, we have varied the contribution of the two processes and asked whether the key features we have identified in our analysis in the presence of feedback between Nodal and tissue packing are general. We found that the reduction in the Nodal range and the faster termination of Nodal signalling we report in the main text are consistent with parameter choices that remove the relay contribution all together and that enhance the relay contribution compared to the parameter set used for our main text results (Fig. SN6a). In both these cases we noted a delay in lefty production in the absence of the feedback (Fig. SN6b) and expansion of the Nodal gradient and reduction in the Nodal concentration near the margin (Fig. SN7), as reported in the main text. To estimate the contribution of the relay mechanism to the total gradient we compared $\lambda_{1/2}(t)$ for the case with relay ($\sigma_N \neq 0$) and the case where we turn relay off ($\sigma_N = 0$) (Figure SN6, c).

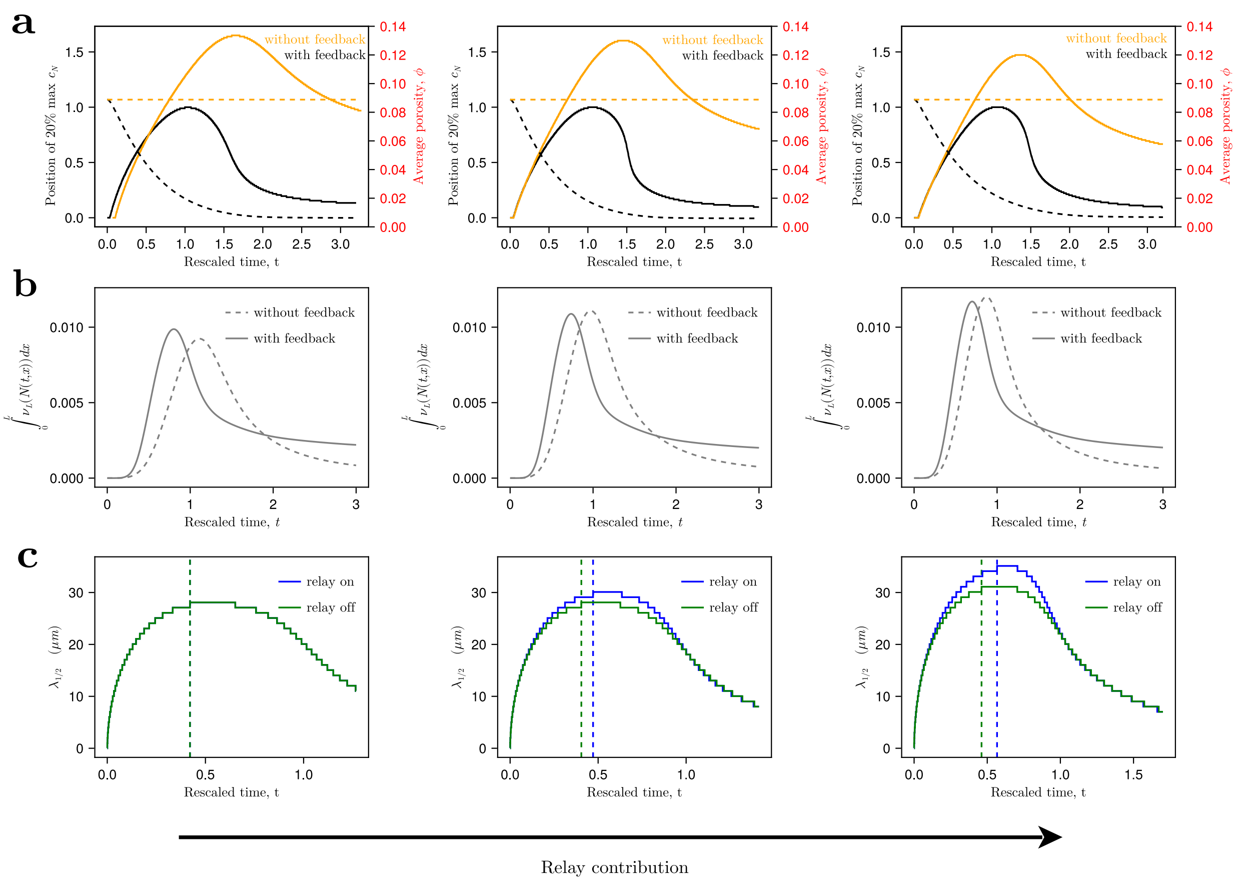

Figure SN6: **(a)** Nodal length scales and averaged porosities from simulations with and without feedback for varying contribution of the Nodal relay component as indicated by the arrow at the bottom of the figure. **(b)** Total Lefty production in the tissue with and without feedback. **(c)** $\lambda_{1/2}(t)$ from simulation with feedback, with and without relay. Parameters: for left column as in main text Fig. 3, 4 but $\sigma_N = 0$, for middle column as in main text Fig. 3, 4, for right column as in main text Fig. 3, 4 but $k_E^{new} = 0.8k_E$ and $\sigma_N^{new} = 1.5\sigma_N$.

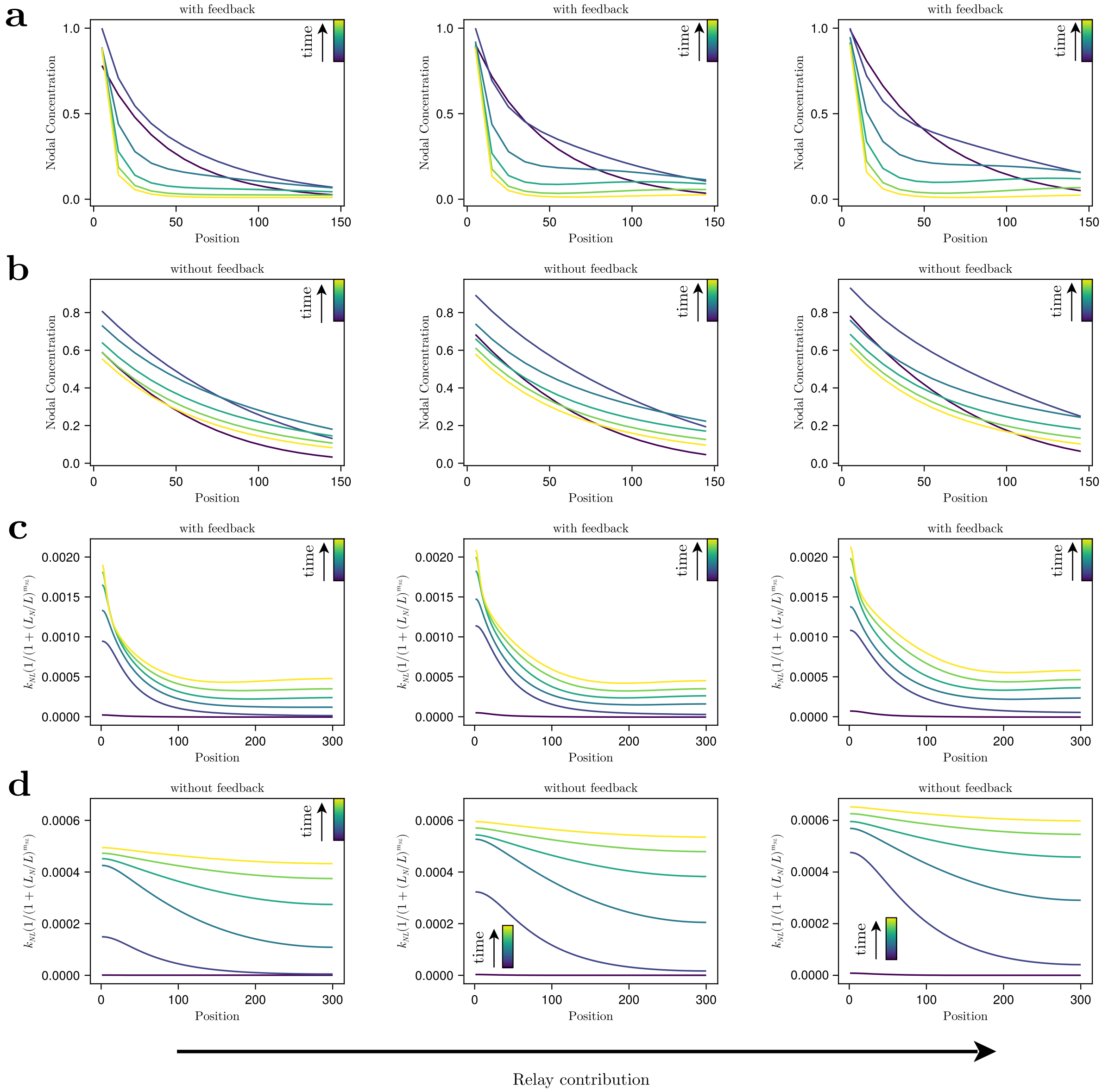

Figure SN7: Nodal concentration profiles with feedback **(a)** and without feedback **(b)** (both scaled to maximum value in (a)), and magnitude of Lefty-induced inhibition with feedback **(c)** and without feedback **(d)**. Time points plotted are $[0.5, 1, 1.5, 2, 2.5, 3]\tau_{WT}$ (from dark blue to yellow). Columns map to parameters that correspond to an increase contribution of relay from left to right as indicated by the arrow at the bottom o Parameters: for left column as in main text Fig. 3, 4 but $\sigma_N = 0$, for middle column as in main text Fig. 3, 4, for right column as in main text Fig. 3, 4 but $k_E^{new} = 0.8k_E$ and $\sigma_N^{new} = 1.5\sigma_N$.

3 Impact of diffusivity and degradation on the morphogen amplitude and decay length

To understand how changes in the effective diffusion, D_{eff} and effective degradation k_{eff} are expected to affect the morphogen amplitude (C_0) and the decay length λ, we consider the minimal diffusion-degradation equation for a morphogen [20]

$$\partial_t C = \partial_x(D_{eff}\partial_x C) - k_{eff}C + s_0\delta(x). \tag{33}$$

with no-flux boundary conditions $\partial_x C|_{x=0} = \partial_x C|_{x=l} = 0$. In the limit $\lambda \ll l$, the (well-known) steady state is

$$C(x) = C_0 e^{-x/\lambda} \tag{34}$$

with the decay length $\lambda = \sqrt{D_{eff}/k_{eff}}$ and amplitude $C_0 = \frac{s_0}{2}\left(k_{eff}\lambda\left(1-e^{-l/\lambda}\right)\right)^{-1} \approx \frac{s_0}{2\sqrt{D_{eff}k_{eff}}}$. Figure SN8 illustrates that, in this simple scenario, an increase in C_0 can be facilitated by a reduction in D. This reasoning indicates that the changes in the GFP-tagged Squint observed experimentally are consistent with the conclusion that the tissue rigidity transition and concomitant decrease in porosity restricts the diffusivity of Nodal ligands (main text Fig. 3m-o and Supplementary Fig. 3k-r).

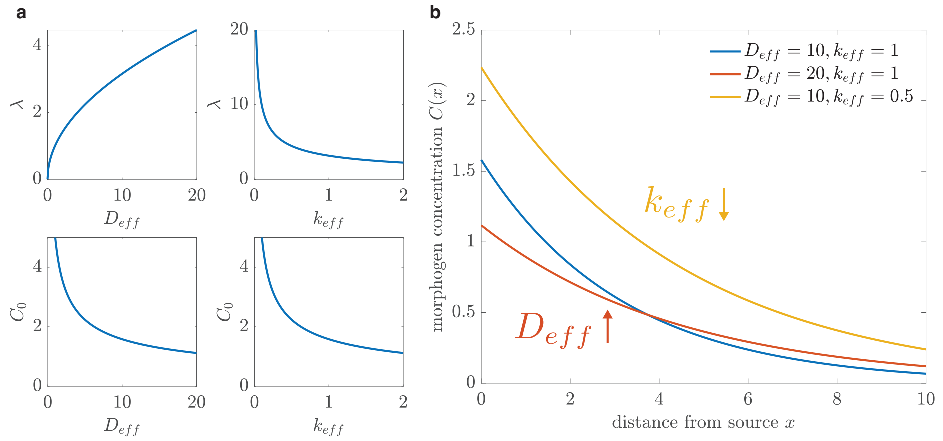

Figure SN8: **(a)** Plots of $\lambda = \sqrt{D_{eff}/k_{eff}}$ and $C_0 = \frac{s_0}{2}\left(k_{eff}\lambda\left(1-e^{-l/\lambda}\right)\right)^{-1}$. **(b)** Halving the degradation or doubling the diffusivity has the same effect on λ, but the amplitude increases in the former case and decreases in the latter. Parameters (if not varied): $s_0 = 10, l = 100$, $D_{eff} = 10$, $k_{eff} = 1$.

4 Simulations of cell networks and cell tilings with varying connectivity and adhesion

In this section we detail the steps followed to construct networks and cell tilings with gradient in adhesion and connectivity along the vertical axis. For the latter, we introduce the soap-bubble Hamiltonian [21, 22], which will become the target function that will lead the optimization process. From the structure of cell-cell contacts, we can derive the rigidity properties of the system. Besides the emerging topological structure of the network of cell-cell contacts, the computation of optimal tilings given a certain surface tension parameter is also used to compute the formation of Tri-cellular junctions.

4.1 Construction of networks with connectivity gradient

The basic network structure is a 2D triangular lattice, in which some disorder in the location of nodes is introduced, which may trigger the appearance of new links, due proximity, or their removal, in case the two nodes get separated more than a threshold beyond which it is considered that they cannot represent a cell-cell contact. Upon this basic structure, to explore how the cell connectivity affects the spatial distribution of the Giant Rigid Cluster (GRC), we generated networks of size 35x35 nodes with a linear connectivity gradient, keeping the global average connectivity fixed. This is achieved by pruning links probabilistically from a totally connected triangular lattice –with geometric disorder, as explained above– with link deletion probability increasing linearly along the vertical axis of the network.

4.2 Construction of in-silico cell-tilings with adhesion gradient

To explore the effects of the adhesion gradient on the GRC localization, we simulate non-confluent tissues as 2D cell arrangements with linearly decreasing adhesion along the vertical axis. The energy of the tissue can be described by a soap-bubble-like Hamiltonian [21, 22]:

$$\mathcal{H} = \alpha \sum_{i<j} w_{ij} + \frac{1}{2} \sum_{i} a_i, \tag{35}$$

where w_{ij} denotes the contact area between cells i and j, a_i is the area of cell i in contact with interstitial fluid, and α is a non-dimensional parameter defined as:

$$\alpha = \frac{\gamma_{cc}}{2\gamma_{cf}} = \cos\left(\frac{\theta}{2}\right), \tag{36}$$

with θ being the angle between the membranes of two cells in contact, γ_{cc} is the cell-cell surface tension, and γ_{cf} is the cell-fluid surface tension. Conservation of cell area is imposed as a boundary condition throughout the optimization process. The above equation establishes a connection between the relation among surface tensions and the angle between two membranes in contact to the fluid, an observable feasible to extract from real systems. Numerical simulations of 2D cell tilings have been performed using the C based software `Surface Evolver` version 2.70 [23]. To start the simulation, the initial configuration needs to be generated, specifying the location of the cells, a primary polygon-like geometry and the initial topology of cell-cell contacts. Later this initial condition will evolve 1/ By increasing the resolution of the perimeter of the cells –thereby achieving realistic geometries– and 2/ Optimizing the global geometry of individual cells and size of cell-cell contacts according to the global soap-bubble-like Hamiltonian as the one in Eq. 35, in a relaxation process towards the desired α value. The action of external pulling -or pushing forces is not considered here and, thereby, we expect the tilings to be in equilibrium with respect the soap-bubble Hamiltonian. To generate random tilings with subcritical target densities ϕ_T, we follow the RSA algorithm [24] as implemented in [15] –further we will introduce the gradient in adhesion:

- Over an a-priori defined square of length L –in units of cell diameter $D = 2R$, where R is the average cell radius– send N' random possible coordinates $\vec{x_1}, ..., \vec{x_{N'}}$.
- The sequential process of generation of random coordinates is subject to a selection criteria: If when generating the k-th random coordinate an already existing coordinate $\vec{x_i}$, $i < k$ is such that $d(\vec{x_i}, \vec{x_k}) < (1-\epsilon)2R$ this coordinate $\vec{x_k}$ is discarded, as it would lead to a large overlapping pair of disks.
- If the number of accepted generated coordinates N ($N \leq N'$) reaches a value such that, $\phi_T \leq N\phi R^2/L^2$, where ϕ_T is the target density, the process stops, since the target density has been achieved.
- If after a long number of iterations, the target density cannot be achieved, we perform a random search along the area identifying possible empty spaces that can be filled using the previous distance conditions.

- We build a collection $C_1, ...C_N$ of disks centered on each of the accepted coordinates $(\vec{x_1}, ..., \vec{x_N})$. For any pair of disks C_i, C_k such that $d(\vec{x_i}, \vec{x_k}) \leq 2R$ we define a contact. Since the presence of overlaps and further changes on adhesion may slightly alter the cell fraction, we may encounter the situation by which the actual achieved density $\phi < \phi_T$. If this happens, we need to generate more disks, refining the halting condition and rewriting it as $\phi \leq (N + \delta)\phi R^2/L^2$ to achieve the desired densities $\phi \approx \phi_T$ after optimization.

Once generated, the seed is stabilized in the hard-disk regime ($\alpha = 1$). Each cell is described by a set of vertices and segments, whose minimum length is controlled by the parameter t. We generate tilings of 26x26 cells. The evolution of the tissue will be simulated as follows.

- Decrease α for each region of the tiling, according to the imposed gradient, in a quasi-static way until the desired α is reached. The gradient is imposed such that the bottom layer has target $\alpha = 0.77$ and the top layer has target $\alpha = 0.95$, values that agree with the measurements in real systems.

  In this step, the mechanism of re-meshing implemented by the `Surface Evolver` acts by alternatively reducing and increasing the resolution of the geometry of the cells. This is done by merging all segments lower than a certain length (t parameter) and subsequently dividing such segments by 2. With this, the optimization algorithm can explore the potential configurations minimizing the global surface energy in an efficient way. According to our simulations, working with very fine grained geometries (low t parameters) all the time may trap the system in local optima. In consequence, we need to introduce sporadically large merging events (large t's) to trigger topological changes. To preserve the high resolution of the simulation but, at the same time, allow topological rearrangements, we apply the re-meshing t-parameter as following a Weibull function:

$$p(t) = \frac{k}{\lambda}\left(\frac{t}{\lambda}\right)^{k-1} e^{-(t/\lambda)^k} \quad .$$

  This allows us to perform most of the re-meshing events around a well defined mean, keeping high resolution, but, from time to time, massive re-meshing events allow topological rearrangements.

- When the targeted α is reached and no changes on the energy are appreciated throughout successive rounds of optimization, we end the process. The resulting configuration is a tiling with the desired α gradient and approximately the target density ϕ_T.

4.3 Rigid cluster analysis

Identification of floppy and rigid areas of the 2D cell-cell contact networks was performed using `pebble.py`, available at: https://github.com/coldlaugh/pebble-game-algorithm/blob/master/pebble.pyx [25].

References

[1] María Almuedo-Castillo, Alexander Bläßle, David Mörsdorf, Luciano Marcon, Gary H. Soh, Katherine W. Rogers, Alexander F. Schier, and Patrick Müller. Scale-invariant patterning by size-dependent inhibition of Nodal signalling. *Nature cell biology*, 20(9):1032–1042, 2018.

[2] Sybren R. De Groot and Peter Mazur. *Non-equilibrium Thermodynamics*. Dover, 1984.

[3] Maria Bruna and S. Jonathan Chapman. Diffusion in Spatially Varying Porous Media. *SIAM Journal on Applied Mathematics*, 75(4):1648–1674, 2015.

[4] Patrick Müller, Katherine W. Rogers, Ben M. Jordan, Joon S. Lee, Drew Robson, Sharad Ramanathan, and Alexander F. Schier. Differential Diffusivity of Nodal and Lefty Underlies a Reaction-Diffusion Patterning System. *Science*, 336(6082):721–724, 2012.

[5] Antonius L. van Boxtel, John E. Chesebro, Claire Heliot, Marie-Christine Ramel, Richard K. Stone, and Caroline S. Hill. A temporal window for signal activation dictates the dimensions of a nodal signaling domain. *Developmental Cell*, 35(2):175–185, 2015.

[6] Yu Chen and Alexander F. Schier. The zebrafish Nodal signal Squint functions as a morphogen. *Nature*, 411(6837):607–610, 2001.

[7] Yu Chen and Alexander F Schier. Lefty Proteins Are Long-Range Inhibitors of Squint-Mediated Nodal Signaling. *Current Biology*, 12(24):2124–2128, 2002.

[8] Graham E. Bell and John Crank. Influence of imbedded particles on steady-state diffusion. *Journal of the Chemical Society, Faraday Transactions 2: Molecular and Chemical Physics*, 70(0):1259–1273, 1974.

[9] Daniel M. Tartakovsky and Marco Dentz. Diffusion in Porous Media: Phenomena and Mechanisms. *Transport in Porous Media*, 130(1):105–127, 2019.

[10] Pierre Recho, Adrien Hallou, and Edouard Hannezo. Theory of mechanochemical patterning in biphasic biological tissues. *Proceedings of the National Academy of Sciences*, 116(12):5344–5349, 2019.

[11] John Crank. *The Mathematics of Diffusion.* Oxford Science Publications. Clarendon Press, Oxford, UK, 2nd edition, 1975.

[12] M. P. Dalwadi, I. M. Griffiths, and M. Bruna. Understanding how porosity gradients can make a better filter using homogenization theory. *Proceedings of the Royal Society A: Mathematical, Physical and Engineering Sciences*, 471(2182):20150464, 2015.

[13] Magnus Röding. Shape-dependent effective diffusivity in packings of hard cubes and cuboids compared with spheres and ellipsoids. *Soft Matter*, 13(46):8864–8870, 2017.

[14] Kristina S Stapornwongkul, Marc de Gennes, Luca Cocconi, Guillaume Salbreux, and Jean-Paul Vincent. Patterning and growth control in vivo by an engineered GFP gradient. *Science (New York, N.Y.)*, 370(6514):321–327, 2020.

[15] Laura Rustarazo-Calvo, Cristina Pallares-Cartes, Adrián Aguirre-Tamaral, Elisa Floris, Maximilian Hingerl, Camilla Autorino, Arif Ul Maula Khan, Bernat Corominas-Murtra, and Nicoletta I. Petridou. Adhesion-driven tissue rigidification triggers epithelial cell polarity. *bioRxiv preprints*, (10.1101/2025.03.18.644006), 2025.

[16] *Sphere packings, lattices and groups / by J.H. Conway and N.J.A. Sloane.* Grundlehren der mathematischen Wissenschaften ; 290. Springer, 1988.

[17] Timo Kuhn, Amit N. Landge, David Mörsdorf, Jonas Coßmann, Johanna Gerstenecker, Daniel Čapek, Patrick Müller, and J. Christof M. Gebhardt. Single-molecule tracking of Nodal and Lefty in live zebrafish embryos supports hindered diffusion model. *Nature Communications*, 13(1):6101, 2022.

[18] Charles Audet and John E Dennis Jr. Mesh adaptive direct search algorithms for constrained optimization. *SIAM Journal on optimization*, 17(1):188–217, 2006.

[19] Johanna E M Dickmann, Jochen C Rink, and Frank Jülicher. Long-range morphogen gradient formation by cell-to-cell signal propagation. *Physical Biology*, 19(6):066001, 2022. by Lewis, 2022.

[20] Ortrud Wartlick, Anna Kicheva, and Marcos González-Gaitán. Morphogen Gradient Formation. *Cold Spring Harbor Perspectives in Biology*, 1(3):a001255, 2009.

[21] Takashi Hayashi and Richard W Carthew. Surface mechanics mediate pattern formation in the developing retina. *Nature*, 431(7009):647–652, 2004.

[22] D. L. Weaire and S. Hutzler. *The Physics of Foams*. Clarendon Press, 1999.

[23] K. Brakke. The surface evolver. *Experimental Mathematics*, 1(2):141–165, 1992.

[24] Einar L. Hinrichsen, Jens Feder, and Torstein Jøssang. Random packing of disks in two dimensions. *Phys. Rev. A*, 41:4199–4209, Apr 1990.

[25] Leyou Zhang, D. Zeb Rocklin, Bryan Gin-ge Chen, and Xiaoming Mao. Rigidity percolation by next-nearest-neighbor bonds on generic and regular isostatic lattices. *Phys. Rev. E*, 91:032124, Mar 2015.
