# Supplementary material for: Tissue rigidity phase transition shapes morphogen gradients
Source: Nat Cell Biol. 2026 May 14;28(6):1191–203. doi: 10.1038/s41556-026-01954-4 (PMC13278970; doi:10.1038/s41556-026-01954-4)
Supplement: Supplementary file 2 — Reporting Summary [file 41556_2026_1954_MOESM2_ESM.pdf]

nature portfolio

Corresponding author(s): Bernat Corominas-Murtra
Zena Hadjivasiliou
Nicoletta I Petridou

Last updated by author(s): March 11 2026

Reporting Summary

Nature Portfolio wishes to improve the reproducibility of the work that we publish. This form provides structure for consistency and transparency in reporting. For further information on Nature Portfolio policies, see our Editorial Policies and the Editorial Policy Checklist.

Statistics

For all statistical analyses, confirm that the following items are present in the figure legend, table legend, main text, or Methods section.

n/a	Confirmed	
☐	☒	The exact sample size (*n*) for each experimental group/condition, given as a discrete number and unit of measurement
☐	☒	A statement on whether measurements were taken from distinct samples or whether the same sample was measured repeatedly
☐	☒	The statistical test(s) used AND whether they are one- or two-sided<br>*Only common tests should be described solely by name; describe more complex techniques in the Methods section.*
☐	☒	A description of all covariates tested
☐	☒	A description of any assumptions or corrections, such as tests of normality and adjustment for multiple comparisons
☐	☒	A full description of the statistical parameters including central tendency (e.g. means) or other basic estimates (e.g. regression coefficient) AND variation (e.g. standard deviation) or associated estimates of uncertainty (e.g. confidence intervals)
☐	☒	For null hypothesis testing, the test statistic (e.g. *F*, *t*, *r*) with confidence intervals, effect sizes, degrees of freedom and *P* value noted<br>*Give P values as exact values whenever suitable.*
☒	☐	For Bayesian analysis, information on the choice of priors and Markov chain Monte Carlo settings
☒	☐	For hierarchical and complex designs, identification of the appropriate level for tests and full reporting of outcomes
☒	☐	Estimates of effect sizes (e.g. Cohen's *d*, Pearson's *r*), indicating how they were calculated

Our web collection on statistics for biologists contains articles on many of the points above.

Software and code

Policy information about availability of computer code

Data collection	ZEN3.3 (Blue Edition, Carl Zeiss), LAS-X (v 3.7.4, Leica Microsystems)
Data analysis	Python (v 3.10.9 - python packages: matplotlib (v 3.7.0), pandas (v 1.5.3 and v 2.2.2), seaborn (v 0.12.2), numpy (v 1.23.5 and v 1.26.4), imageio (v 2.34.2), scikit-image (v 0.24.0), scipy (v 1.14.0)), Fiji (v 2.3.0, ImageJ2), Imaris (v 10.1, Oxford Instruments), Cellpose (v 2 and v 3), GraphPad Prism (v 10) and CellRanger (v 7.1.0, 10x Genomics) were used.

For manuscripts utilizing custom algorithms or software that are central to the research but not yet described in published literature, software must be made available to editors and reviewers. We strongly encourage code deposition in a community repository (e.g. GitHub). See the Nature Portfolio guidelines for submitting code & software for further information.

Data

Policy information about availability of data

All manuscripts must include a data availability statement. This statement should provide the following information, where applicable:

- Accession codes, unique identifiers, or web links for publicly available datasets
- A description of any restrictions on data availability
- For clinical datasets or third party data, please ensure that the statement adheres to our policy

The data supporting these findings are provided in the Source Data. The single cell sequencing datasets generated in this study are publicly available with the

following Gene Expression Omnibus (GEO) accession codes: GSE299074, GSM9032491, GSM9032492. The lefty1 coding sequence used to design the smFISH probes is available under accession code ENSDART00000019196.7.

Research involving human participants, their data, or biological material

Policy information about studies with human participants or human data. See also policy information about sex, gender (identity/presentation), and sexual orientation and race, ethnicity and racism.

Reporting on sex and gender	*Use the terms sex (biological attribute) and gender (shaped by social and cultural circumstances) carefully in order to avoid confusing both terms. Indicate if findings apply to only one sex or gender; describe whether sex and gender were considered in study design; whether sex and/or gender was determined based on self-reporting or assigned and methods used. Provide in the source data disaggregated sex and gender data, where this information has been collected, and if consent has been obtained for sharing of individual-level data; provide overall numbers in this Reporting Summary. Please state if this information has not been collected. Report sex- and gender-based analyses where performed, justify reasons for lack of sex- and gender-based analysis.*
Reporting on race, ethnicity, or other socially relevant groupings	*Please specify the socially constructed or socially relevant categorization variable(s) used in your manuscript and explain why they were used. Please note that such variables should not be used as proxies for other socially constructed/relevant variables (for example, race or ethnicity should not be used as a proxy for socioeconomic status). Provide clear definitions of the relevant terms used, how they were provided (by the participants/respondents, the researchers, or third parties), and the method(s) used to classify people into the different categories (e.g. self-report, census or administrative data, social media data, etc.) Please provide details about how you controlled for confounding variables in your analyses.*
Population characteristics	*Describe the covariate-relevant population characteristics of the human research participants (e.g. age, genotypic information, past and current diagnosis and treatment categories). If you filled out the behavioural & social sciences study design questions and have nothing to add here, write "See above."*
Recruitment	*Describe how participants were recruited. Outline any potential self-selection bias or other biases that may be present and how these are likely to impact results.*
Ethics oversight	*Identify the organization(s) that approved the study protocol.*

Note that full information on the approval of the study protocol must also be provided in the manuscript.

Field-specific reporting

Please select the one below that is the best fit for your research. If you are not sure, read the appropriate sections before making your selection.

☒ Life sciences ☐ Behavioural & social sciences ☐ Ecological, evolutionary & environmental sciences

For a reference copy of the document with all sections, see nature.com/documents/nr-reporting-summary-flat.pdf

Life sciences study design

All studies must disclose on these points even when the disclosure is negative.

Sample size	No sample size calculation was performed. Sample size is different in each experiment. All samples sizes are reported in figure legends. Sample size was chosen based on our previous experience and the work of other groups using zebrafish embryos as a model system.
Data exclusions	No data were excluded from the analysis
Replication	All attempts at replication were successful. At least more than three independent experiments were performed. This information is also stated in the figure legends.
Randomization	No randomization methods were used to determine how samples/organisms were allocated.
Blinding	No blind allocations during data collections and/or analysis was relevant to the study.

Reporting for specific materials, systems and methods

We require information from authors about some types of materials, experimental systems and methods used in many studies. Here, indicate whether each material, system or method listed is relevant to your study. If you are not sure if a list item applies to your research, read the appropriate section before selecting a response.

Materials & experimental systems

n/a	Involved in the study
☐	☒ Antibodies
☒	☐ Eukaryotic cell lines
☒	☐ Palaeontology and archaeology
☐	☒ Animals and other organisms
☒	☐ Clinical data
☒	☐ Dual use research of concern
☒	☐ Plants

Methods

n/a	Involved in the study
☒	☐ ChIP-seq
☒	☐ Flow cytometry
☒	☐ MRI-based neuroimaging

Antibodies

Antibodies used

Rabbit anti pSMAD2/3(8828S, Cell Signalling, 1:1000 - MAb - Clone D27F4, Lot 8), Schauer et al., Elife, 2020
Mouse anti beta-catenin (C7202, Sigma Aldrich, 1:100 - MAb - Clone 15B8, Lot 089M4857V),
Mouse anti-E-Cadherin (Cat. No. 610181, BD Biosciences, 1:200 - Lot 4283007), Pollock 2021 Prion
Goat anti-rabbit Alexa 647 (A21244, Invitrogen, 1:500 - Lot 2247991)
Goat anti-mouse Alexa 647 (A21235, Invitrogen, 1:500 - Lot 2272554)
Goat anti-mouse Alexa 488 (A11001, Invitrogen, 1:500 - Lot 2220848)
Sheep alkaline phosphatase anti-digoxigenin antibody (11093274910, Roche, 1:2000 - Lot 32871922)

Validation

Antibodies were validated by the specificity of their location and have been previously validated in zebrafish:
- anti-pSMAD2/3: Van Boxtel et al., Developmental Cell, 2015; Schauer et al., Elife, 2020; Lord et al., Elife, 2021; https://www.cellsignal.com/products/primary-antibodies/phospho-smad2-ser465-467-smad3-ser423-425-d27f4-rabbit-monoclonal-antibody/8828
- anti-beta-catenin: Schauer et al., Elife, 2020; https://www.sigmaaldrich.com/DE/en/product/sigma/c7207
- anti-E-cadherin: Pollock et al., Prion 2021; Lord et al., Elife, 2021; https://www.bdbiosciences.com/en-de/products/reagents/western-blotting-and-molecular-reagents/purified-mouse-anti-e-cadherin.610181?tab=citations_references
- anti-rabbit Alexa 647: https://www.thermofisher.com/antibody/product/Goat-anti-Rabbit-IgG-H-L-Cross-Adsorbed-Secondary-Antibody-Polyclonal/A-21244
- anti-mouse Alexa 647: https://www.thermofisher.com/antibody/product/Goat-anti-Mouse-IgG-H-L-Cross-Adsorbed-Secondary-Antibody-Polyclonal/A-21235
- anti-mouse Alexa 488: https://www.thermofisher.com/antibody/product/Goat-anti-Mouse-IgG-H-L-Cross-Adsorbed-Secondary-Antibody-Polyclonal/A-11001
- anti-DIG: Schauer et al., Elife, 2020; https://www.sigmaaldrich.com/DE/en/product/roche/11093274910

Animals and other research organisms

Policy information about studies involving animals; ARRIVE guidelines recommended for reporting animal research, and Sex and Gender in Research

Laboratory animals

Danio rerio zebrafish strains wildtype A2B2, Tg(mezzo:eGFP), MZwnt11/slb-tx226, MZoep-tz257, MZlefty1-a145/lefty2-a146, Gt(ctnna-citrine)ct3a at adult (7-20 months) and embryonic stages (until 7 hours post fertilization) were used.

Wild animals

The study did not involve wild animals

Reporting on sex

Female and male adult fish were used for natural mating. The sex of the embryos used is unknown.

Field-collected samples

The study did not involve filed-collected samples

Ethics oversight

All animal experiments were carried out according to the guidelines of the Committee for Animal Welfare and Institutional Animal Care and Use (IACUC) under EMBL's Policy on the Protection and Welfare of Animals Used for Scientific purposes (IACUC code 21-010_HD_NP).

Note that full information on the approval of the study protocol must also be provided in the manuscript.

Plants

Seed stocks	*Report on the source of all seed stocks or other plant material used. If applicable, state the seed stock centre and catalogue number. If plant specimens were collected from the field, describe the collection location, date and sampling procedures.*
Novel plant genotypes	*Describe the methods by which all novel plant genotypes were produced. This includes those generated by transgenic approaches, gene editing, chemical/radiation-based mutagenesis and hybridization. For transgenic lines, describe the transformation method, the number of independent lines analyzed and the generation upon which experiments were performed. For gene-edited lines, describe the editor used, the endogenous sequence targeted for editing, the targeting guide RNA sequence (if applicable) and how the editor was applied.*
Authentication	*Describe any authentication procedures for each seed stock used or novel genotype generated. Describe any experiments used to assess the effect of a mutation and, where applicable, how potential secondary effects (e.g. second site T-DNA insertions, mosiacism, off-target gene editing) were examined.*
